# Natural rhizobial diversity helps to reveal genes and QTLs associated with biological nitrogen fixation in common bean

**DOI:** 10.3934/microbiol.2017.3.435

**Published:** 2017-06-08

**Authors:** Olaya Muñoz-Azcarate, Ana M González, Marta Santalla

**Affiliations:** Departamento de Recursos Fitogenéticos, Grupo de Biología de Agrosistemas, Misión Biológica de Galicia-CSIC. P.O. Box 28. 36080 Pontevedra, Spain

**Keywords:** *Phaseolus vulgaris*, *Rhizobium*, symbioses, biodiversity, Quantitative Trait Loci

## Abstract

Common bean is one of the most important crops for human feed, and the most important legume for direct consumption by millions of people, especially in developing countries. It is a promiscuous host legume in terms of nodulation, able to associate with a broad and diverse range of rhizobia, although the competitiveness for nodulation and the nitrogen fixation capacity of most of these strains is generally low. As a result, common bean is very inefficient for symbiotic nitrogen fixation, and nitrogen has to be supplied with chemical fertilizers. In the last years, symbiotic nitrogen fixation has received increasing attention as a sustainable alternative to nitrogen fertilizers, and also as a more economic and available one in poor countries. Therefore, optimization of nitrogen fixation of bean-rhizobia symbioses and selection of efficient rhizobial strains should be a priority, which begins with the study of the natural diversity of the symbioses and the rhizobial populations associated. Natural rhizobia biodiversity that nodulates common bean may be a source of adaptive alleles acting through phenotypic plasticity. Crosses between accessions differing for nitrogen fixation may combine alleles that never meet in nature. Another way to discover adaptive genes is to use association genetics to identify loci that common bean plants use for enhanced biological nitrogen fixation and, in consequence, for marker assisted selection for genetic improvement of symbiotic nitrogen fixation. In this review, rhizobial biodiversity resources will be discussed, together with what is known about the loci that underlie such genetic variation, and the potential candidate genes that may influence the symbiosis' fitness benefits, thus achieving an optimal nitrogen fixation capacity in order to help reduce reliance on nitrogen fertilizers in common bean.

## 1. Introduction

Common bean (*Phaseolus vulgaris* L.) is cultivated worldwide, constituting a staple in developing countries in East Africa and South America, and also in some regions of Asia, where it is the main source of protein [1]. In some of them, common bean can account for up to 20% of the total daily protein intake per person [2],[3]. In Europe, there has been a notable increase in common bean consumption in past years, due to a greater demand for healthy and functional food [4], and the current trend of vegetarian diets in Central Europe and the United Kingdom, in which beans and other pulses are included as meat substitutes. It is accepted that domestication of common bean from wild forms took place several thousand years ago in two main and independent centers of diversification, Mesoamerica (Mexico and Central America) and the Andes (Peru, Bolivia, and Northwest Argentina), resulting in two highly differentiated gene pools [5]. From these centers, the crop was spread all over the American continent, and, after the first voyages of Columbus (1492), common bean was brought to Europe. Both common bean gene pools spread widely in all parts of Europe with very complex pathways of dissemination that included several introductions from various regions of the Americas, combined with direct exchanges between European and other Mediterranean countries [6] and adaptation to European soils and climate conditions [3],[7],[8],[9]. During the five centuries since the introduction of common beans into Europe, many varieties evolved under diverse environments and farmer preferences, to provide dry seeds or fresh pods [10], thus the loss of variation might have been less than previously suspected. What is more, hybridization that occurred in Europe between the Andean and Mesoamerican gene pools probably had a significant impact on the maintenance of the overall level of genotypic diversity [9].

Common bean can establish symbiotic interactions with both rhizobia (the *Rhizobium*-legume symbiosis, RL) and arbuscular mycorrhizal (AM) fungi, leading to the formation of nitrogen-fixing nodules and phosphate acquiring mycorrhiza [11]. Both symbiotic interactions play a vital role in ecosystems and sustainable crop production, and are central for efforts to decrease dependence on commercial fertilizers. The intensive application of N fertilizers over the last century has perturbed the N cycle, by leaching excess N fertilizers to watercourses and the emission of pollutant NO_x_ gases to the atmosphere [12],[13]. Common bean is generally known as a weak Nitrogen (N) fixer in comparison with other grain legumes [14],[15]. Therefore, application of N fertilizers in bean fields is recommended to achieve higher yields. Selection in the 20^th^ century based on common bean varieties with the best performance in highly mechanized monoculture systems is often reported to be the cause of the current relatively low Symbiotic Nitrogen Fixation (SNF) [16]. However, it has been reported that the climbing and indeterminate common bean varieties consistently have higher nodulation and SNF abilities, compared with most bush-type cultivars. These greater abilities are attributed to the relatively longer period of fixation during the growth cycle in climbing type cultivars [17]–[20]. Miranda and Bliss [21] reported that selection for high levels of SNF, especially when performed in low-fertility soils, might result in genetic gains in common bean breeding populations. Bliss [14] also discussed that the level of N fixation can vary significantly among common bean genotypes, and argued that reports of insufficient levels of N fixation were often based on observations with only a few genotypes, and were conducted with unsuitable N fixation measurement assays. Thus, proper characterization and evaluation of common bean germplasm collections as sources of adaptive alleles, and their utilization in breeding for enhanced SNF, are often limited or neglected. In addition to genetic background, several abiotic factors can greatly influence the SNF ability of common bean. Deficiencies of phosphorus (P), potassium (K), and sulfur (S) have been reported as environmental SNF-limiting factors, which may influence number and weight of nodules [22],[23]. Direct impacts of P, K and (or) S deficiencies on nodules might be due to their influence on physiological and metabolic processes in nodules [23],[24]. Other important environmental factors affecting SNF are salinity and different soil water conditions [25],[26].

As a consequence, selection of best adapted common bean cultivars and most effective fixing rhizobial strains in each association is a must in order to maximize SNF [19]. Therefore, the study of rhizobia's natural diversity is a source of ecological information about symbioses, as it allows for the definition of host preferences and strain predominance, but, most importantly, since it provides the source for efficient strains to be used as inoculants in agricultural fields [18].

Breeding programs for improved SNF in common bean have been developed, resulting in the release of high N fixing Mesoamerican cultivars, promoting the development of cropping systems that are less dependent on N chemical fertilizers [27]. However, sustained success in developing Andean cultivars with enhanced SNF has been elusive. The availability of superior genotypes with higher N-fixation ability supports the idea that SNF in common bean may be improved through breeding efforts. In this sense, the advances in genetics and genomics resources of common bean and the high degree of synteny between this crop and its legume crops relatives can be exploited so as to understand complex traits associated with SNF, leading to the discovery of new genes or Quantitative Trait Loci (QTLs), as well as to improve genetic maps and develop molecular markers for Marker Assisted Selection (MAS). In this perspective, rhizobial biodiversity resources and the genes or QTLs of adaptive importance for RL interaction will be discussed, in order to accelerate the development of common bean cultivars with enhanced SNF.

## 2. Exploring the Natural Diversity of Rhizobia Nodulating Common Bean in Natural and Agricultural Soils

Diversity of the common bean nodule rhizobia has been extensively studied across many countries, showing that in the centers of origin, and in Latin America in general, where beans have been grown for several thousand years, a huge diversity of bean nodulating rhizobia is found. These native strains are very competitive for nodule occupancy, but, in general, they show a low N fixing efficiency [18]. Nevertheless, their efficiency can be increased with adequate selection processes and agronomical practices. Intensive selection programs of highly efficient rhizobial strains have been carried out in the last few years in Brazil, as one of the main dry bean-producing countries, in order to select elite inoculants [28],[29].

The rhizobia classification has undergone major changes and revisions and several novel species have been described. The family *Rhizobiaceae* comprises seven main genera harboring plant-associated species, *Rhizobium*, *Neorhizobium*, *Allorhizobium*, *Agrobacterium*, *Ensifer* (syn. *Sinorhizobium*), *Shinella* and the genus *Ciceribacter*. From 1996, the classification and molecular characterization of rhizobial species has been based mainly on the comparative analysis of gene sequences encoding for the 16S rRNA ribosomal subunit, a housekeeping gene [30]. However, events of recombination in these 16S rRNA genes have been reported [31] and genomes of rhizobia may lose or gain plasmids or genomic islands [32]. As a result, 16S rRNA sequencing may not be that informative for rhizobial taxonomy, and alternate phylogenies may be constructed using other housekeeping genes as well as the *nod* and *nif* genes, related to the symbiotic process [31]. In order to solve the taxonomic uncertainties concerning the plant-associated members of the *Rhizobiaceae* family, Mousavi et al. [32] performed Multilocus Sequence Analysis (MLSA) of 100 strains of the family *Rhizobiaceae* and 16 rhizobial strains from other rhizobial families, using four housekeeping genes namely 16S rRNA, *atpD* (ATP synthase F1, beta subunit), *recA* (recombinase A), and *rpoB* (RNA polymerase, beta subunit). The delineation of the new genus *Pararhizobium* and 13 new species combinations were proposed [32]. *Rhizobium* genus is a heterogeneous group accommodating two major sub-clusters: *R. tropici* and *R. leguminosarum.* The genus name *Rhizobium* encompasses 56 species; however, it is not a proper name for all, since some of them are phylogenetically interspersed among members of other genera in *Rhizobiaceae*. For instance, *R. oryzae* did not group with *Rhizobium* members according to several Multi Locus Sequence Analysis (MLSA) studies [33]. Another controversial group is the genus *Ensifer*; data obtained recently indicate that *Ensifer adhaerens* and “*Sinorhizobium morelense*” are not heterotypic synonyms, but represent separate species [34]. There are two main databases specialized in the phylogeny, taxonomy and diversity of the *Rhizobiaceae* family: http://edzna.ccg.unam.mx/rhizobial-taxonomy/ (CCG, UNAM, Cuernavaca, Mexico) [35], and http://www.rhizobia.co.nz/taxonomy/rhizobia [36], in which new rhizobial species are regularly added or reclassified.

Up to date, five genera and 19 species nodulating common bean have been described, including *R. leguminosarum* bv. *phaseoli*, *R. etli* bv. *phaseoli*, *R. gallicum* (bv. *phaseoli* and bv. *gallicum*), *R. giardinii* (bv. *phaseoli* and bv *giardinii*), *R. lusitanum* [37] and *R. tropici* [38]–[41], and, more recently, *Pararhizobium giardinii* [32]), *R. ecuadorense* [42], *R. vallis, R. leucaneae* [43], *R. mesoamericanum* [44], *R. mongolense, R. oryzae* [45] , *R. freirei* [46], *R. rhizogenes, R. azibense* [47], *R. acidisoli* [48], *R. hidalgonense* [49], as well as *Ensifer meliloti* [50],[51], *E. medicae* [52], *E. americanus* [53], *Bradyrhizobium* spp. and the beta-proteobacteria *Burkholderia* [52],[54],[55]. Despite this high promiscuity, *R. etli* bv. *phaseoli* was found to be the predominant mycrosymbiont in both the Mesoamerican and the Andean common bean's centers of origin [1],[3],[40],[56], and in many different areas across the whole world such as Southern and Central Europe [57],[58], Tunisia [52], Central and West Africa [59],[60], Ethiopia [55],[61], Indonesia [59] and in Northeast China [62]. However, recent taxonomic studies reclassified some of the strains previously considered as *R. etli* into the *R. phaseoli* group or other species of common-bean rhizobia [63],[64].

**Figure 1.**
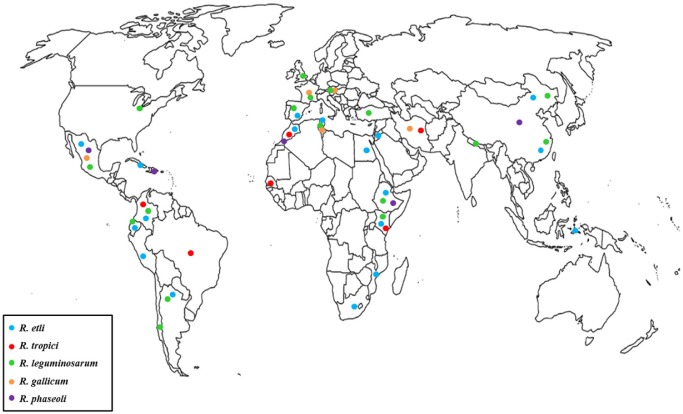
Distribution of the predominant rhizobium species in common bean in each country.

In the tropics or in areas with high temperatures and/or acidic soils, *R. tropici* replaces *R. etli* as the preferred symbiont for common bean. *R. tropici* shows a good tolerance to both constraints, even when beans from Mesoamerican cultivars are used as trap hosts [65],[66]. In these areas, *R. tropici* has been proved to be far more competitive for nodulation than *R. etli*, blocking the nodulation of *R. etli* [67]. Two types of *R. tropici*, A and B have been clearly distinguished that seem to be diverging lineages sharing a common symbiotic plasmid [56],[68], although there are some *R. tropici* strains with intermediate characteristics between A and B that do not belong to any of them [56],[66],[69]. Recently, accumulated phylogenetic data supported the reclassification of *R. tropici* strains belonging to type A into the new species *R. leucaneae* [43]. Recent genome analyses of *R. tropici* CIAT899 and two commercial strains of *R. leucaneae* revealed that these strains are well equipped to cope with low pH, high temperatures, and with oxidative and osmotic stress [70],[71]. Figure 1 and Table 1 summarize the areas for several important rhizobia species, which are described below.

### 2.1. American continent

In Mexico, other bacteria apart from *R. etli* bv. *phaseoli* have been isolated in bean nodules (*R. leguminosarum* bv. *phaseoli* [72]). Actually, the genetic diversity of rhizobial isolates in Mexico is so large that it could serve as the basis to propose new species [18],[56], although most of the identified ones correspond to *R. etli*, *R. gallicum* bv. *phaseoli* [73] or *R. leguminosarum* clusters [73]. According to the population genetic analyses performed by Silva et al. [72], there is a high genetic differentiation between the *R. etli* and *R. gallicum* strains isolated from common bean in Mexico, with no genetic exchange between them. In saline and/or alkaline soils, however, *Ensifer* *americanus* was the predominant symbiont for common bean [73]. The isolates of *E. americanus* were classified in a subcluster different to the *R. etli* strains isolated in the same study [73]. Also, a novel rhizobium species, *R. hidalgonense*, has recently been isolated from nodules of common bean cultivated in acidic conditions in the Hidalgo state of Mexico [49], and classified attending to the sequences of housekeeping genes *atpD*, *glnII* and *recA*. This novel strain showed a high similarity (between 92 and 94%) with the type strains of related *Rhizobium* bean-nodulating species, including *R. etli* and *R. phaseoli.*

**Table 1.** Predominant rhizobium species isolated from common bean nodules in each country.

Specie	Country	Method for classification	References
***R. etli***	Mexico, Colombia, Ecuador, Peru	16S rRNA gene sequencing	[39]
	Argentina	16S rRNA gene sequencing, RFLP of *nodC* gene	[3]
	Spain	MLEE, RFLP and partial sequencing of the 16S rRNA genes, RFLP of symbiotic genes	[57]
	Austria	RFLP of 16S rRNA and PCR of *nifH* genes	[58]
	Tunisia	16S rRNA, RFPL of *nifH* and *nodC g*enes	[52]
	Morocco	RAPD fingerprinting of housekeeping and *nodC* genes	[100]
	Egypt	16S rRNA gene sequencing	[103]
	Ethiopia	AFLP fingerprinting and MLSA on 16S rRNA, *nodC* and *nifH* genes	[55]
	Kenya	Sequencing of *nif* genes	[105]
	South Africa and Mozambique	16S rRNA gene and other housekeeping genes, symbiotic genes	[108]
	Jordan	*nifH* and *nod* genes	[110]
	Indonesia	16S rRNA gene sequencing	[59]
	Northeastern China	16S rRNA gene, and other housekeeping genes sequencing	[62]

***R. tropici***	Colombia	16S rRNA gene sequencing	[39]
	Brazil	16S rRNA gene sequencing	[69]
	Senegal and Gambia	SDS-PAGE, RFLP of 16S rRNA gene	[60]
	Kenya	Sequencing of *nif* genes	[105]
	Morocco	16S rRNA gene sequencing	[98],[99]

***R. leguminosarum***	Mexico	MLSA of 16S rRNA, and other housekeeping genes *nifH* and *nodC* genes,	[72],[73]
	Ecuador	Phenotypic analysis and PCA of Box AIR-PCR banding patterns	[74]
	South-central Chile	RFLP of 16S rRNA and *nodC* genes	[87],[88]
	USA	16S rRNA gene sequencing, biochemical and morphological characters	[89]
	England	16S rRNA gene sequencing, biochemical and morphological characters	[43]
	Spain (Northwest)	Analysis of ITS regions in housekeeping genes, sequencing of *nodC* genes; sequence analysis of housekeeping genes	[95],[96]
	France	16S rRNA gene sequencing	[40]
	Tunisia	16S rRNA gene sequencing, RFPL of *nifH* and *nodC* genes	[52]
	Ethiopia	AFLP fingerprinting and MLSA on 16S rRNA, *nodC* and *nifH* genes	[55]
	Kenya	SDS-PAGE, RFLP of 16S rRNA gene	[105]
	Turkey	16S rRNA and other housekeeping genes and, *nif* and *nodA* genes sequencing	[109]
	Northeastern China	Partial sequencing of 16S rRNA, and other housekeeping genes	[62]
	Nepal	16S and 23S rRNA, *nodC* and *nifH* genes sequencing	[118]

***R. gallicum***	Mexico	MLSA of 16S rRNA , and other housekeeping genes, *nifH* and *nodC* genes	[72]
	France	16S rRNA gene sequencing	[40]
	Austria	RFLP of 16S rRNA and PCR of *nifH* genes	[58]
	Tunisia	16S rRNA gene sequencing, RFPL of *nifH* and *nodC* genes	[52]
	Iran	RFLP of 16S rRNA gene	[111]

***R. giardinii***	France	16S rRNA gene sequencing	[40]

***R. phaseoli***	Mexico	MLSA of 16S rRNA, and other housekeeping genes *nifH* and *nodC* genes	[63]
	Morocco	RAPD fingerprinting of housekeeping and *nodC* genes	[100]
	Ethiopia	AFLP fingerprinting and MLSA on 16S rRNA, *nodC* and *nifH* genes	[55]
	Dominican Republic	RAPD fingerprinting of housekeeping and *nodC* genes	[85]

In Ecuador and Peru, rhizobia isolated from bean nodules have a high diversity, and may be divided into clusters different from the Mexican isolates [74], which suggests coevolution, or, at least, host selection [75]. It should be noted that in Ecuador, apart from *R. etli*, *R. leguminosarum* has been identified as another predominant strain in common bean nodules [74]. Common bean gene pool of Peru-Ecuador is unique, as it is originated in the borders between the two main centers of diversification [76], thus, studies of microsymbionts from this region are receiving increasing attention. As a result, *Rhizobium ecuadorense* sp. nov., an indigenous N-fixing symbiont of the Ecuadorian common bean, has recently been characterized, based on DNA-DNA hybridization, 16S rRNA gene sequence phylogeny and MLSA of three other housekeeping genes [42]. *R. ecuadorense* strains have been classified in the *R. phaseoli*/*R. etli*/*R. leguminosarum* phylogenetic clade, but showed unique biochemical and physiological properties, constituting a separate subclade.

Studies of common bean rhizobial diversity in other Mesoamerican countries are scarce. Nicaraguan common bean cultivars were reported to improve their productivity when inoculated with a commercial strain, highlighting the potential of these cultivars for N fixation efficiency [77], and the need for screening native rhizobial strains. In Guatemala, the *R. leguminosarum* strain CIAT632 was isolated from active nodules of wild and cultivated Mesoamerican common bean cultivars [78], although its predominance in common bean nodules in native Guatemalan soils has not yet been tested.

In Colombia, apart from *R. etli, R. tropici* is predominant; additionally, the reference strain CIAT899 was isolated in acid soils [38]. Later on, in 1995, a phylogenetic comparison of common bean isolates from Mexico and Colombia identified a genotypically diverse group of Colombian rhizobial strains strongly differentiated from the Mexican *R. etli* group, according to the Multilocus Enzyme Electrophoresis (MLEE) of the 16S rRNA gene sequence; all these 27 isolates were identified as Colombian native strains, although it is not clear if they belong to *R. etli*, *R leguminosarum* or *R. tropici* group [79].

In Northwest Argentina, in the Andean center of origin, *R. etli* was also found to be predominant in nodules of wild bean varieties [80], by using plasmid profile and DNA fingerprinting. In this study, 31 out of the 35 soil isolates presented the *R. etli* 16S rDNA allele, and phylogenetic similarity between pairs of isolates ranged from 71 to 99% [81]. Later, the diversity of rhizobial strains nodulating common bean in the same area was described using a collection of 400 isolates of rhizobia recovered from local soils and nodules [3]. The isolates were characterized by the analysis of 16S rRNA, *nifH* and *nodC* genes, and most isolates were identified as *R. etli* or *R. leguminosarum*, with a minor presence of *Ensifer fredii*, *E. saheli*, *S. terangae* and *Mesorhizobium loti*. Aguilar et al. [81] also analyzed the *nodC* genes in several *R. etli* strains from different countries (Mexico, Ecuador, Peru, Bolivia, Northwest Argentina and Colombia) belonging to the centers of common bean diversification. They found that the different *nodC* alleles from these American strains had varying predominance in each center of diversification, suggesting coevolution of common bean and rhizobia in the centers of host genetic diversification.

In Brazil, *R. tropici* and *R. leucaneae* are also the predominant species in common bean nodules, due to the good tolerance of these species to the acid soils of this country [1],[66],[67], even though *R. etli* is the most abundant in Brazilian soils [82]. In a phylogenetic study, the Brazilian *R. etli* strains were found to be closely similar in 16S rRNA sequences and in *nodABC* and *nifH* RFLP-PCR profiles to the Mexican CFN42 strain, and quite distinct from *R. etli* and *R. leguminosarum* strains from Europe [1], thus supporting the hypothesis that Brazilian common bean cultivars and their rhizobia are of Mesoamerican origin, and could have arrived in Brazil at pre-colonial times. In this study, *R. tropici* reference strains and *Rhizobium* sp. strains were clustered together in the same group at a level of similarity of 51%, and the 43 Brazilian *R. etli* strains constituted a large cluster along with the reference strain CFN42. *R. tropici* may have already been present in Brazilian soils, and, due to its tolerance to acidic soils and high temperature, it became the predominant microsymbiont of common bean. Supporting this hypothesis, *R. tropici* strains were recently isolated and characterized from wild common bean Brazilian varieties [83], with high genetic similarity to the reference strain CIAT899, and also with a similar symbiotic efficiency. *R. leucaneae* is abundant in the Brazilian Cerrados soil, and is frequently used as an inoculant due to its capacity to nodulate common bean and fix N under stressful environmental conditions [65]. Two other new species previously classified as *R. tropici*, *R. freirei* and *R. paranaense*, were isolated from common bean nodules in Brazil [46],[84] and both proved to be very effective for N fixation, constituting an important source of commercial inoculants in the tropics, along with *R. tropici* and *R. leucaneae*. *R. freirei* was placed within the *R. tropici* phylogenetic group, based on the analysis of *recA*, *atpD* and *rpoB* gene sequences.

Caribbean islands, located along the trade routes between the Americas and Europe, played a crucial role in the exchange of common bean seeds and their endosymbionts, although only few studies are available about strains native of these islands. The Hispaniola Island, comprising the countries of Haiti and Dominican Republic, was the first stopover in the voyages of Columbus between America and Spain. The analysis of genomes of strains nodulating common bean in coastal and inner regions on this island showed that they were almost identical to American *R. etli* strains (recently reclassified as *R. phaseoli*) [85]. In Cuba, beans are widely cultivated all over the country and are at the core of the daily diet. Four *Rhizobium* species were isolated and identified from common bean nodules in Cuban soils: unclassified *Rhizobium* sp., *R. etli*, *R. radiobacter* and *R. pisi* bv. *viciae* (previously classified as *R. leguminosarum*). *R. pisi* was the most often isolated species, (20%) in addition to *R. etli* (10%) [86].

In Chile, the predominant species that nodulates common bean is *R. leguminosarum* [87], although the presence of three other species of rhizobia, *R. leucaneae*, *R. tropici* and *R. etli*, in common bean rhizosphere or nodules have recently been identified according to the *nodC* PCR-RFLP pattern in this South-Central region [88]. *R. leguminosarum* was also found to have the highest genetic diversity in both acidic and alkaline soils in central and South-Central locations.

In the United States of America (USA), common bean was rarely inoculated until recently, and, as a result, few studies regarding this issue have been found. In the state of Minnesota, rhizobia were recovered from bean nodules and characterized according to reference strains. Analysis of 16S rRNA gene sequences revealed that half the organisms tested were most similar to *R. leguminosarum*, with a small presence of *R. etli*-like strains [89].

### 2.2. European continent

In Europe, rhizobial species with high genomic similarity to those at the common bean centers of origin have also been isolated, suggesting that they were present in the surface of bean seeds when these where brought out from America [1],[57],[90]. However, promiscuity of common bean to nodulation with bacterial strains is much higher in introduced areas, where bean functions as a trap-host plant [91],[92]. Segovia et al. [39] proposed that *R. etli* strains in the surface of the bean seeds could have transferred their symbiotic plasmid to *R. leguminosarum* strains present in European soils, allowing *R. leguminosarum* to recognize and nodulate common bean. Later, *R. leguminosarum* would have transferred the Sym plasmid with specific genes for common bean nodulation to *R. gallicum* and *R. giardinii* [40]. In most areas where common bean was introduced, cooccurrence of several strains is common, although with different symbiotic efficiencies.

In Portugal, a species named *R. lusitanum* was identified after isolation and molecular analysis of strains from several soils [37]. Afterwards, the diversity of 179 bean rhizobial isolates from Northwest Portugal was investigated. Five of the six phylogenetic groups were placed within *R. lusitanum*, and the remaining one was placed within *R. tropici* group, according to the RAPD analysis of housekeeping genes [93]. However, according to *nodS* gene sequencing, four *R. lusitanum* strains were placed within *R. etli* cluster, showing a similarity of 91–99% to the *nodS* sequence of the reference strain CFN42; the remaining strain was placed within the same cluster of *R. tropici*, and its *nodS* sequence was 100% identical to that of the CIAT899 reference strain. These classifications suggest that *R. lusitanum* was the recipient of the genes for symbiosis from both *R. etli* and *R. tropici*.

In Spain, five different rhizobial species, *R. etli*, *R. leguminosarum*, *R. gallicum*, *R. giardinii* and *S. fredii* (=*Ensifer fredii*) isolated from a single soil, were found to nodulate common bean [57]. Martínez-Romero and Caballero-Mellado [56] have also revealed the existence of one of the most genetically diverse collections of *Rhizobium* isolates from common bean nodules, being *R. etli* the predominant species. Later on, Velázquez et al. [94] identified the same five rhizobia species in bean nodule isolates from two different regions, Andalucía and León in the South and Northwest of Spain, respectively, using the staircase electrophoresis method for identification. However, García-Fraile et al. [95] found that common bean is nodulated preferentially by *R. leguminosarum* in a wide region in Northern Spain, the major dry bean producer region. Also, the inoculation of native *R. leguminosarum* strains from this area in common bean local varieties produced similar grain yield and number of pods than the N-fertilized control [96]. The genetic analysis showed that these *R. leguminosarum* isolates carry the same alleles for the *nodC* genes as the American strains of this species. These results suggest that *R. etli* could have had difficulty in persisting in the soils of Northern Spain when it was brought from America, probably because of the climatic conditions [95]. The symbiotic genes of this species might have been transferred, after the arrival of common bean, to strains of *R. leguminosarum* already present in Northern Spanish soils, and become better adapted. In the arid and saline soils of Lanzarote, on the Canary Islands, most of the isolates from common bean were identified as *E. meliloti* and *E. fredii*, according to the analysis of *nodC* sequences [51].

Other three *Rhizobium* species, *R. leguminosarum*, *R. gallicum* and *R. giardinii*, have also been isolated in France and Austria, and probably represent bacteria that pre-existed in European soils before common bean was introduced [40],[58]. *R. leguminosarum* is also the predominant species in common bean nodules and soils in Croatia, although these data come only from five out of the twenty-three soil samples analyzed, since most soil samples in this study contained no indigenous common bean rhizobia [97]. The authors suggest cropping history and N fertilization for common bean as the possible origin of this absence of rhizobial presence in Croatian soils. Nevertheless, also in this case, inoculation of common bean with indigenous Croatian *R. leguminosarum* resulted in better nodulation and symbiotic efficiency than inoculation with the reference commercial strain.

### 2.3. African continent

Morocco is the main producer of green bean in Africa, and common bean was brought to Africa via Spain and Portugal after the colonization of the Americas. Therefore, American rhizobial strains harbored in these seeds were also distributed in Africa. In Morocco, *R. tropici* type B and other rhizobia (identified as *R. leguminosarum* and pseudo-*Agrobacterium*) were isolated from bean nodules; some of them were also highly salt-tolerant [98],[99]. Salinity is an increasing problem in North Africa and in the Mediterranean, affecting RL symbioses and common bean productivity. In Morocco, common bean is cultivated in saline soils and its symbiosis depends on the presence or application of osmotolerant strains in these soils. Faghire et al. [100] identified 32 osmotolerant rhizobial strains from common bean collected in Moroccan soils, and classified them attending to the RAPD patterns of the housekeeping genes *rrs*, *recA*, *atpD* and the symbiotic gene *nodC*. The most abundant species were placed either within the *R. etli* or the *R. phaseoli* phylogenetic group, and a second group was identified as *R. gallicum*. The remaining strains belonged to the *R. tropici* phylogenetic group, with almost 100% identity to CIAT899 [100]. In another study, four *Bulkholderia phymatum* strains were also isolated from bean nodules grown in alkaline soils in Morocco [54]; all of them were able to fix N efficiently.

In Tunisia, the most abundant rhizobial species in bean nodules were *R. gallicum*, *R. etli* and *R. leguminosarum*, each of them representing between 20 and 29 % of the isolates [52], according to the RFLP analysis of 16S rRNA, *nifH* and *nodC* gene fragments. Four other minor groups of rhizobia were classified as *R. giardinii* and *Ensifer spp.* One of these isolated *R. gallicum* Tunisian strains, 23C2, was recently reclassified within the novel species *R. azibense* [47]. The 16S rRNA gene of this strain showed a 99% similarity with *R. gallicum* and *R. mongolense* reference strains, but phylogenetic analysis based on the sequences of housekeeping genes *recA*, *atpD*, *dnaK* and thrC showed that this strain represents a separate genomic group [47]

Also, in alkaline and/or saline Tunisian soils, *Ensifer* species as *E. fredii*, *E. meliloti* and *E. americanum* [101],[102] were found to be the predominant symbionts of common bean, and they were phylogenetically related to the above-mentioned *Ensifer* strains isolated and identified in Spain [51] and Mexico [73] for these types of soil.

In Egypt, *R. etli* and *R. gallicum* were isolated from different soils, and were able to nodulate common bean, displaying cultivar-dependent symbiotic efficiency [103]. *R etli* represented about half of the isolates, characterized by the analysis of 16S rRNA sequences, whereas *R. gallicum* had a minor presence. Salinity and alkaline soils are also a major problem contributing to the low productivity of common bean in Egypt; the same authors identified new *R. etli* strains highly tolerant to these environmental stresses [104]. These results are consistent with the above-mentioned for bean nodule isolates from Tunisia [52].

In Sub-Saharian locations, the high acidic soils and high temperatures favor the prevalence of *R. tropici* in common bean nodules, as was reported in Kenya [105], Senegal and Gambia in West Africa [60]. In Kenya, several rhizobia were isolated from two soils with pHs of 4.5 and 6.8 and characterized on the basis of their host ranges and sequencing of *nif* genes. Most isolates were similar to *R. etli* and *R. leguminosarum* bv. *phaseoli*, while the predominant strain in acidic soils was likely to be *R. tropici*, although their characterization was presumptive, since little molecular evidence was gathered [105]. Later on, the symbiotic efficiency of native rhizobia nodulating common bean was assayed, showing a higher efficiency than the reference strain CIAT899 [106]. Commercial inoculants used in Western Kenya still contain exotic cultures from the USA [107], which may not be well adapted to local conditions; therefore, the identification of these strains is essential.

Ethiopian soils, as happens in neighboring Kenya, also harbor natural populations of rhizobia forming symbioses with common bean [61], although the diversity pattern is different from that reported in the latter. Based on Multilocus Enzyme Electrophoresis, the majority of the Ethiopian genotypes were genetically related to the type strain of *R. leguminosarum*. However, from analysis of 16S rRNA genes, most of them were placed in *R. etli* group. The possible reason for this might lie in the transfer and recombination of the 16S rRNA gene from the introduced *R. etli* to the local *R. leguminosarum*, as suggested for Europe rhizobial populations [39],[40],[93]. Additionally, a more extensive screening of native common bean rhizobial strains in Ethiopian soils revealed that the majority of the isolates belonged to the species *R. etli*, *R. leguminosarum* and *R. phaseoli*, with a minor presence of *R. leucaneae* and *R. giardinii*, based on the sequencing of symbiotic (*nodC* and *nifH*) and housekeeping genes (*recA*, *glnII*, *rpoB*, and 16S rRNA) [55].

The diversity and phylogeny of root-nodule bacteria from common bean has also been studied recently in South Africa and Mozambique [108], using phylogenies of both symbiotic and housekeeping genes (16S rRNA and others). The analysis of *nifH* and *nodC* symbiotic genes allowed for the classification of most isolates into the *R. etli* group, with a small proportion of *R. tropici*-like genomes. Again, this classification was inconsistent with the phylogram of the housekeeping genes, which is an indication of lateral transfer among the *Rhizobium* isolates. *R. leucaneae*, belonging to the *R. tropici* group, was also isolated in Eastern Cape Province of South Africa, in very stressful acidic conditions, as in the Cerrados region in Brazil [65].

### 2.4. Asian continent

In Turkey, historical records indicate that cultivation of common bean started much later that in Europe, about 250–300 years ago [109]. In the Central Black Sea Region, 30 rhizobial isolates obtained from common bean have been classified according to the sequencing of housekeeping genes as 16S rRNA and the symbiotic genes *nifH* and *nodA* [109]. Half of the isolates were identified as *R. leguminosarum*, while the others were placed in either the group of *R etli* bv. *phaseoli* (8 out of 30 isolates) or *R. phaseoli* (6 out of 30). The phylogenetic analysis based on sequence data from *nodA* and *nif* genes showed that all rhizobial species in this study had the same haplotypes as the *R. etli* CFN42 reference strain, suggesting a further support for the lateral transfer of *R. etli* Sym plasmid amongst Turkish common bean nodulating isolates.

In Jordan, *R. etli* represented 80% of rhizobial species, in a collection of 30 isolates from common bean cultivated in 16 different locations, after analysis of *nod* and *nif* genes. A second group, comprising the remaining 20%, was identified as *R. tropici* [110]. *R. etli* was also the predominant strain in Egypt, and neighboring Jordan; taking into account the trade relationship between both countries, *R. etli* strains from both countries are likely to share a common origin [103].

Iran seems to constitute a separate biogeographical region from that of Turkey and Jordan, regarding common bean rhizobial diversity. Fifty-three isolates were sampled from common bean root nodules cultivated in different locations in Iran, and half of them were placed into either *R. gallicum* or *R. tropici* type B phylogenetic groups, according to the RFLP analysis of the 16S rRNA gene [111]. Despite the fact that an accurate identification of these strains is still pending, they seem to be native of Iran, and showed a good symbiotic effectiveness as well as some plant-growth promoting traits such as P solubilization and production of auxins and siderophores when inoculated in common bean [112].

As for East Asia, most of the studies about common bean rhizobia have been conducted in China, considered to be a secondary center of diversity for common bean [113]. Common bean was introduced from Latin America to China 600 years ago [114], and it has been widely cultivated throughout the country ever since. As a result, a huge diversity in common bean rhizobia can be encountered there, differing from one region to another. *R. leguminosarum* and *R. etli* were dominant in different ecoregions [62],[115],[116],[117], with varied relative abundances. The analysis of the *nodC* and *nifH* genes in all the *Rhizobium* strains isolated in China, along with the predominance of *R. etli*, suggest that these species may have an American origin [117]. There was also a significant presence of *Bradyrhizobium* spp., *R. phaseoli* and *Ensifer fredii* in common bean nodules [115],[117]. However, *Bradyrhizobium* seems to be an opportunistic symbiont for common bean, since it formed ineffective nodules when inoculated separately [117]. In addition, two new rhizobial species were isolated from wild rice in China, *R. mongolense* and *R. oryzae*, which were also able to effectively nodulate common bean [45].

*R. etli*, *R. leguminosaum* and *R. phaseoli* were also the most abundant species in common bean in Nepal, characterized by the partial sequencing of 16S and 23S rRNA, and *nodC* and *nifH* genes. The diversity in each of the seven sampled fields is strongly affected by soil pH and temperature; however, the predominant species in all soils was *R. leguminosarum* [118].

### 2.5. Oceania

Common bean has been grown in some Oceanian countries as Australia and New Zealand since the arrival of Europeans, and it is commonly cultivated in rich-N soils, fertilized with N or supplied with commercial inoculants [119],[120]. However, symbiotic relationships between common bean and native rhizobia remain poorly characterized despite their importance [121]. In Australia, several strains of natural rhizobia have been isolated from native legumes or soil samples, and *E. meliloti* [121], *R. leguminosarum* and *R. tropici* were identified [122].

## 3. Bacterial Molecular Determinants of Specificity in Rhizobia-common Bean Interactions

The large differences in symbiotic effectiveness within strains nodulating common bean were suggested to result from the coadaptation and coevolution of common bean cultivar and bacteria [4]. Some biogeographical analyses of common bean-rhizobia symbioses mentioned before, in which a parallel genetic variation has been reported, support this idea of coevolution in the centers of origin [18]. Coevolution has also been proposed as an explanation for the specificity of the interactions. However, rhizobia and common bean phylogenies are not parallel, because this is a weak and non-obligate symbiosis [123], and so the idea of coevolution is still to be proved.

The specificity of rhizobia-legume interaction is determined by both bacterial and host plant compounds, although common bean presents a high versatility for the association with different rhizobia sepecies and genera [92]. Several bacterial molecules are know to be involved in this specificity, including Nod factors, cell wall polysaccharides, and type I and III secreted proteins [124],[125],[126]

Cell wall polysaccharides include exopolysaccarides (EPS), lipopolysaccharides (LPS) and β-glucans [124],[125]. The interactions between rhizobial surface polysaccharides and plant lectins (carbohydrate-binding receptors present in legume root hair surfaces) have long been considered to be determinant in host recognition by rhizobia [126]. EPS are released to the environment, and are the main surface polysaccharides related to symbiosis specificity, as alterations in rhizobial EPS can lead in some cases to impairment in symbiosis [125],[127]. EPS are heteropolymers consisting in repeating units of monosaccharides substituted with non-carbohydrate residues, and a high diversity in their chemical structure has been described, which may be the cause of their involvement in symbiotic specificity [127]. Biosynthesis of EPS in rhizobia is a complex process regulated at both transcriptional and post-transcriptional levels and influenced by several environmental conditions, including hyperosmotic stress, temperature, oxygenation and pH, and also nutrients concentrations (nitrogen, sulfur, and phosphate) which may alter the recongition between symbionts [127],[128]. LPS, composed by an oligosaccharide and a lipid that binds the molecule to the outer membrane, is another cell wall determinant of rhizobial identity. LPS acts as a suppressor of plant defense responses, key to allow the progression of bacteria inside the infection thread, and it has been proposed to be involved in the symbiosis specificity. However, the precise role of LPS in symbiosis remains unclear, and its effect in nodulation varies depending on the species [125],[129].

However, most authors agree that the key components of host specificity in rhizobia are *nod* genes [124],[125],[126],[130]. These genes are included in the Sym plasmids, which are specific for each biovar (a group of strains nodulating the same legume). *Nod* genes encode for the Nod factors (NF) released by rhizobia, as a response to plant flavonoids, chemical signals exudated by the legume that initiate the nodulation process [124],[125],[130]. Daidzein, coumestrol, naringenin, genistein, liquiritigenin, and isoliquiritigenin isoflavones were the major components of common bean *cv.* Rab39 extract, responsible for inducing expression of the *nod* genes of *Rhizobium tropici*, *R. etli*, and *R. leguminosarum* bv. *phaseoli* [131]. The NF synthesized by the rhizobia are perceived by legume plants and cause developmental responses such as the initiation of cell divisions in both cortex and pericycle as well as major deformations of root hair extension, leading to bacterial invasion. NF are lipochitooligosaccharides, complex molecules with a basic structure composed by an oligosaccharide, to which a fatty acid substitute is bond. The synthesis of this basic skeleton depends on *nodA*, *nodB* and *nodC* genes [130],[131]. NodC is the first enzyme in the pathway, and synthesizes a chitooligosaccharide using N-acetyl-glucosamine as substrate. NodB is the responsible of deacylate the non-reducing end of the oligosaccharide, which is subsequently N-acylated by NodA. To this basic structure several chemical groups can be added (including oligosaccharides, methyl, acetyl and sulphide groups, fatty acids, etc), originating a great variety of NF. The addition of these substitutions is encoded by other *nod* genes (*nodH*, *nodPQ*, *nodL*, etc), and depending on the rhizobial strain, different groups can be added [131]. This variety of NF constitutes the major determinant of host specificity in rhizobia, since, depending on their structure and substitute chains, they are perceived by different legume species [125],[126],[130],[131],[132]. Therefore, Sym plasmids, rather than core genomes of rhizobia, might have coevolved with the host plant [133]; this host selection of rhizobial genotypes seems to be a general phenomenon in most legume-rhizobia symbioses [134],[135].

The presence of compatible rhizobia species and their corresponding NF is generally sufficient to trigger rhizobial infection and nodule development in common bean and other legumes [136]. Chemical structures of NF of most common bean-nodulating rhizobium strains have been characterized, revealing that they all have a common basic structure essential to nodulating common bean [137]–[141]. *R. tropici* CIAT899 strain has a broad host range, as it is able to synthesize the greatest diversity of NF found amongst the rhizobia species [141]. The explanation may be found in the complex regulatory mechanisms of NF production in this strain. Five *nodD* genes (regulators of NF production) have recently been sequenced and characterized in CIAT899, using *nodD* mutant strains; it was proposed that each one of the *nodD* genes perform different roles depending on the host plant and the environment [142]. Also in this study, almost 40 different NF were identified in *R. tropici*, and the synthesis of NF was enhanced under salt stress, suggesting that this diversity of NF might play a role in the tolerance of *R. tropici* to abiotic stresses [142]. In another study on *R. tropici* CIAT899, three different *nodA* genes were found in the Sym plasmid, which may also contribute to its broad host range [70].

Biotic and abiotic stresses strongly influence the selection of bacterial strains. Several abiotic stresses such as salinity and osmotic stress [142], drought [143], heavy metals [144], high temperature or low pH [145] have been found to affect either the production of NF or EPS by the bacterial partner or the perception of these compounds by the legume host. Plant pathogens often compete with rhizobia for colonization, and can inhibit their growth in the rhizosphere, their NF production, or their attachment to the legume root hairs [146]. Therefore, for each niche, common bean has chosen the best adapted strains. This is supported by the fact that diversity of rhizobial strains is much lower inside nodules of common bean than in the root surface [75]. In another recent population genomics study, the sequencing and comparison between genomes and Sym plasmids from rhizobial species, isolated from rhizosphere and common bean nodules, confirmed that all these strains coexist in both environments with very low genetic recombination across their core genomes [147]. On the other hand, Sym plasmids of rhizobial strains are extremely similar, with high rates of recombination, and do not appear to have coevolved with the chromosome or with other plasmids. In fact, a phylogenetic classification based on RFLP analysis of *nodC* and *nifH* genes revealed close relationships among common bean symbionts, regardless of their 16S-rRNA-based classification [148]. Lateral gene transfer of the Sym genes appears to be the most likely explanation for the phylogenetic incongruence between Sym and housekeeping genes such as 16S rRNA. This genetic transfer might have occurred across rhizobial species and, in some cases, also across *Rhizobium* and *Ensifer* genera, which may explain why some *Ensifer* strains are able to nodulate common bean in certain situations. Eventually, these transfer events have resulted in recombinant Sym plasmids, as is the case of a common bean nodulating *E. fredii* strain (isolated in Granada, Spain), which harbors a conjugative Sym plasmid assembled from two *R. etli* Sym plasmids and the core genome of *E. fredii* [149].

Thus, all the bacterial genes needed for SNF have been identified. Now, exciting progress is being made in elucidating the common bean plant's contribution to this mutually beneficial interaction, with the identification of crucial signal transduction genes early involved in the response to *Rhizobium*. In addition, the advent of current high-throughput technology provides valuable data that contribute to understanding the metabolic activity during bacterial N fixation. A genome-based study of the metabolic activity in N fixation, involving *R. etli* bacteroids located at the root nodules of common bean, revealed 415 proteins and 689 upregulated genes that orchestrate N fixation [150]. A change of metabolic activity in these enzymes, as a result of gene deletion, induced different effects in N fixation, which is in agreement with observations made in *R. etli* and other *Rhizobiaceae*. Also, a proteomic study on *Rhizobium tropici* PRF81 [151] allowed for the identification of proteins involved in the responses to heat stress, then revealing the diversity of adaptation mechanisms presented by this thermotolerant strain.

## 4. Genomic Common Bean Resources for Functional Analysis of the Rhizobia-common Bean Interaction

One way to discover adaptive alleles for SNF is to research large germplasm collections and to use association genetics so as to identify loci of interest. Both of these genetic approaches are based on the use of biodiversity and may eventually help in identifying the genes that plants use to respond to environmental challenges. In legumes, two wild species, *Medicago truncatula* and *Lotus japonicus*, as well as the cultivated soybean (*Glycine max*) have been adopted as models for genomic studies. The high degree of synteny among these model plants and their legume crops relatives, such as common bean, can be exploited to improve genetic maps and identify candidate genes for symbiotic genes [152]. In addition, research efforts have recently focused on developing the genomics resources, reference genome sequences and catalogues of mutant collections for nodulation efficiency, etc., required to carry out the identification of candidate genes within the common bean species.

### 4.1. Common bean genes involved in nodulation efficiency and comparison with relative legumes

Genomes of many legume species such as *Lotus japonicus*, [153], *Medicago truncatula* [154], soybean [155], chickpea [156] and common bean [157],[158] have been entirely sequenced. Sequence conservation and genetic colinearity between common bean and soybean [159],[160], which diverged from a common ancestor approximately 19 million years ago [155],[161], allows for genomic information to be leveraged from one species to the other [162].

Comparative genomics has allowed for map-based cloning of genes required for nodulation in legumes; one example is the nodulation receptor kinase (*NORK*) gene that is required for both bacterial and fungal symbiosis [163]. *NORK* orthologs were located in the syntenic regions of four legume species (*Medicago*, alfalfa, *Lotus* and pea), and showed high conservation levels (similarity between 87 and 97%) [164]. Schmutz et al. [155] discovered 52 soybean nodulation genes, of which sixteen unigenes were seen to be expressed abundantly in common bean root tissues. Strong syntenic relationships between corresponding genomic regions were found for 20 SNF genes, where soybean and chickpea were the most closely related, followed by soybean and common bean; common bean and *Medicago* were, on the other hand, the most distantly related pair [165]. Other genomic analyses led to the characterization of 191, 92, 65 and 91 soybean, *Medicago*, *Lotus* and common bean orthologous and paralogous to functionally describe nodulation genes, respectively [166]. Common bean also controls the nodule number via an inbuilt signalling mechanism known as the Autoregulation of Nodulation (AON), which is mediated by novel peptide hormones called CLAVATA/ESR-related (CLE) [167]; hypernodulation is primarily controlled by the shoot [168] in both soybean and common bean species [169]. Hastwell et al. [170] identified 84 and 44 CLE peptide-encoding genes in soybean and common bean, respectively, and only three of the 44 genes identified in common bean did not have an apparent ortholog in soybean. Ferguson et al. [171] identified central components in the AON pathway of common bean, such as *PvNARK* which encodes a leucine-rich repeat receptor like protein kinase receptor (LRR-RLK). These kinases play an essential role in the signal transduction required in the early events of nodule formation. NF released by rhizobia are perceived by the LRR extracellular domain of the LRR-RLK in legume root cells, resulting in the initiation of the complex signaling cascade that ultimately leads to the formation of nodules [172]; in addition, it might also regulate root nodule numbers [171]. In soybean, AON involves long-distance signaling, requiring the interaction of RHIZOBIA-INDUCED CLE peptides (*RIC1/RIC2*), with NODULE AUTOREGULATION RECEPTOR KINASE (NARK) in the leaf and the subsequent inhibition of nodulation via the production of the nitrate-induced CLE peptide (*NIC*), which interacts with NARK in the root resulting in a nitrate-induced inhibitor [173]. In common bean, the homologous of *RIC* and *NIC* genes, *PvRIC1*, *PvRIC2* and *PvNIC1*, were identified [174], and as happened in soybean, *PvRIC1* genes were expressed in inoculated common roots at early stages of rhizobial infection, while *PvRIC2* was expressed at later time points in pre-fixing and mature nodules.

### 4.2. Transcriptome analysis of symbiosis

Through an examination of common bean mutants defective in nodulation and transcriptome dynamics, the root hair regulatory pathway activated in response to rhizobia inoculation or NF treatment was characterized.

Thus, the major advances originate from the discovery of plant genes that, if mutated, would affect the symbiotic function of the plant. By predominantly using mutagenesis with the chemical ethyl methyl sulfonate (EMS) followed by selection of the second mutant generation (M2), loss-of-function mutants were isolated for a large range of legumes (notably pea, soybean, *Lotus* and *Medicago* [175]–[178] including common bean (Table 2). Such mutants demonstrated that plant genes are essential for symbiotic success. Through the examination of this research in legumes, it was evidenced that *NF-YC* gene family has been involved in the development of determinate nodules, and that the *NF-YC1* subunit is required for nodule organogenesis and rhizobial infection, as well as for the activation of cell cycle genes at early stages of the symbiotic interaction in common bean [179].

Transcriptomic datasets were released in order to perform a comprehensive analysis of the evolution of legume protein coding genes controlling the nodulation process. Ramírez et al. [180] sequenced 21,026 ESTs from various cDNA libraries (among other N-fixing root nodules and P-deficient roots) derived from the Negro Jamapa genotype. Approximately 10,000 ESTs were identified and at least 15 ESTs showed very high expression ratios in nodule-leaf and nodule-stem. These ESTs were identified as proteins for nodulation (leghemoglobin, nodulin 30 and early nodulin 55-2). In addition, a total of 41 independent common bean tentative consensus sequences (TCs) were differentially expressed in response to two *R. etli* strains of varying nodulation efficiency, of which nine were confirmed to differentially accumulate in efficient interactions [181]. Galeano et al. [182] developed a set of 313 intron-based markers for nodulation genes or genes expressed during nodulation. Quiceno-Rico et al. [183] cloned and characterized two cDNAs (*PvuTRX1h* and *PvuASH1h*) that encoded polypeptide homologs of trithorax group proteins, and demonstrated that *PvuTRX1h* is abundant at the early stages of nodule development, whereas *PvuASH1h* functions at the stages of highest N-fixing activity of the nodules. Montiel et al. [184] identified nine members of the *Rboh* gene family and found that *PvRbohB* accumulated abundantly in shoots, roots, and nodules. Islas-Flores et al. [185] found that the *PvRACK1* increased during nodule development at 12–15 days post-inoculation. Barraza et al. [186] showed that trehalose accumulation in common bean is triggered by *PvTRE1* downregulation, leading to an increased bacteroid number, nodule biomass, and nitrogenase activity and resulting in improved SNF. Dalla Via et al. [187] identified 2,606 genes from common bean that were differentially regulated at early stages of its interaction with *Rhizobium etli*. Formey et al. [136] identified new microRNAs and their corresponding targets in common bean that may function in the regulation of early nodulation events. The key regulatory role of *miR172/AP2* during the rhizobia N-fixation symbiosis has been reported for soybean and common bean [173],[188],[189],[190].

**Table 2.** Description of common bean mutants induced by EMS (Ethyl Methane Sulphonate) affected at different stages of nodule development.

**Cultivar**	**Mutant name (gene)**	**Main characteristics^1^**	**References**
**RIZ30**	NOD238 (*sym-2*)	Fix–, IN, small nodules, poor pod fertility	[212],[213]
	NOD125 (*sym-1*)	Nod–	[214],[215],[216]

**RIZ36**	NOD109 (*sym-2*)	Fix–, delayed nodulation, poor pod fertility	[215],[216]
	R69 (*nie*)	Ineffective nodulation, mr epistatic Park & Buttery, 1997; to nts, non allelic to sym-2 Ineffective nodulation, mr epistatic Park & Buttery, 1997; to nts, non allelic to sym-2 IN, MR epistatic to *nts*, non allelic to *sym-2*	[215],[216]
	R99 (*nnd-2*)	nod–, MR, epistatic to *nts* and *nie*	[215],[216]

**OAC Rico**	R699 (*nie*)	nod+/fix–, Myc–, IN, MR	[214],[217],[218]
	R99 (*nnd-2*)	nod–, Myc–,	[168],[219]
	R32 (*nts*)	NTSN, MR, delayed maturity, and slightly lower yield	[168],[218],[219]

**Swan Valley**	SV145	Nod–, IN, NTSN, MR	[218]

^1^nod–: no nodules; nod+: few nodules; fix–: no N fixation; myc–: resistant to mycorrhiza colonisation; IN: ineffective nodulation (non-functional nodules); NTSN: nitrate tolerant supernodulation; MR: monogenic trait and recessive gene.

### 4.3. Mapping the genetic basis of symbiotic variation

Genetic variability for SNF and associated traits within common bean has been widely reported [119],[191]–[194]. Rennie and Kemp [195] showed that cultivars with a longer vegetative growth period have a longer N fixation time and in consequence were high N fixers. Seventeen cultivars of common bean were investigated for symbiotic compatibility with 10 genetically diverse strains of bean rhizobia [196]; the strain USDA9001 was the most productive strain in terms of seed yield, and common bean varieties Italian Barlotti and BAT271 showed the highest values for both N fixation and plant weight. Rodiño et al. [197],[198] characterized several genotypes of common bean for their ability to fix N with native *R. leguminosarum* and the reference strain *R. tropici* CIAT899. A correlation between shoot growth, N fixation rate and nodulation was observed, suggesting that growh performance and plant yield are dependent on SNF. Recently, Polania et al. [199] investigated the phenotypic variability in SNF ability of several common bean varieties under drought stress in a field harboring native *Rhizobium* strains. Four common bean cultivars showing both higher SNF efficiency and grain yield were selected, that could be used in common bean breeding programs for drought tolerance.

QTL analysis could be combined with a candidate gene approach to seek underlying genetic mechanisms for N use efficiency and SNF in common bean, and there are contrasting materials that can be used as sources of QTLs. Most bean breeding programs do not routinely select SNF because phenotyping is time-consuming, especially when large populations have to be screened. Furthermore, some of the main agronomic traits for breeding such as early flowering and determinate plant architecture are closely related to low N fixation efficiency [15],[195]. Another handicap for the genetic improvement of SNF is its genetic complexity. SNF and nodulation related traits in common bean have a complex inheritance with the involvement of multiple genes [200]–[205]; additionally, their expression is significantly affected by environmental factors [206]. Several plant traits including nodulation, photosynthesis, biomass accumulation, and partitioning of photo-assimilates to the nodules are involved in SNF [207]. A non-additive genetic variation explains nodule number and shoot weight, while additive genetic variation was found for nodule weight in common bean [208]. Overall, all these factors limit the genetic enhancement of SNF, and therefore, the understanding of the genetic architecture of SNF in terms of genomic regions and/or genes involved is critical to expand knowledge of its genetic control.

The first QTL study on nodule number (NN) trait in common bean was performed by Nodari et al. [200], who reported four genomic regions for NN in a Recombinant Inbred Line (RIL) population derived from a cross between BAT93 and Jalo EEP558 varieties, and accounting for 50% of the phenotypic variation. Tsai et al. [201] and Souza et al. [202] screened the same RIL population under low and high N levels, finding new seven and five QTLs for NN under low and high N conditions, accounting for 34 and 28% of total phenotypic variation, respectively. DNA sequence comparison of markers closely linked to these QTLs allowed for the identification of some potential candidate genes. One of these genes encodes an auxin-responsive transcription factor, and might explain differences in N fixation ability between climbing and bushy cultivars [201]. One limitation of these studies is that NN is only an indirect measure of SNF, and in fact, it only gives an idea of nodulation efficiency, not of N fixation itself. Asfaw et al. [207] reported that QTLs for leaf chlorophyll content (SPAD) were the most consistent across environments. A RIL population derived from G2333 × G19839 was investigated for NN, nodule weight, SPAD, shoot dry weight, biomass N, and seed N under both field and greenhouse conditions, with QTLs for NN, nodule dry weight and Ndfa at harvest [203]. Overlapped QTLs for SPAD in the greenhouse and in the field were detected on Pv01 and Pv07. Two major QTLs for percent N fixed and total plant N fixed, contributed to 17 and 21% of phenotypic variation, respectively, and two candidate genes were detected underlying these QTLs: an auxin-responsive transcription factor, and AP2/ERF-domain-containing transcription factor. The former is associated with differences in growth, yield and N accumulation, while the latter with total amount of symbiotic N fixed. Farid [208] used single-nucleotide polymorphism (SNP) markers in a RIL population derived from a low × high-SNF navy bean population, and detected 42 QTLs for %Ndfa (Nitrogen derived from the atmosphere) on Pv01, Pv07 and Pv08. The QTL mapped in Pv08 accounted for up to 17% of the phenotypic variation; while other QTL located onin Pv07, accounting for 14% of the variation, was significantly associated with protein content. A correlation between %Ndfa and late flowering and maturity was detected, empowering the ability of plant to carry out N fixation [208]. Heilig et al. [209] identified 17 unique QTLs associated with SNF traits, most of them located in three large clusters on Pv01 (4 QTLs), Pv06 (6 QTLs), and Pv08 (6 QTLs), in a RIL from the cross of Puebla 152 × Zorro grown in the field and greenhouse under N-free conditions. Many of the QTLs were also associated with candidate genes expressed (transcription factors, transferases, and receptors involved in sensing rhizobacteria) in the nodules and roots.

A Genome Wide Association study (GWAs) focused on SNF traits in an Andean diversity panel of 259 common bean genotypes was carried out by Kamfwa et al. [205], and 11 significant SNPs were found closely linked to Ndfa in the shoot at flowering and in the seed. Several QTLs for Ndfa were confirmed on Pv03 and Pv07, and one SNP on Pv09 was associated with significant QTLs for Ndfa in the seed and shoot, SPAD, shoot biomass, and %N in shoot biomass. This close genetic association between N fixation and growth performance has to be taken into account in marker-assisted breeding programs in common bean for SNF. Three genes (*Phvul.007G050500, Phvul.009G136200* and *Phvul009G231000*) were identified as candidate genes for Ndfa [205]. The *Phvul.009G136200* gene on Chromosome (Chr) 09 codes for a LRR-RLK. The candidate gene *Phvul.007G050500* on Chr07 also encodes for a LRR-RLK, with a role in nodule development [210]. The sequence of *Phvul.009G23231000* showed a high correlation with that of genes coding for calmodulin, which are calcium transporting proteins, in *Arabidopsis thaliana* (TAIR) and *Medicago* (NCBI) [211]. Calmodulin transporters mediate the calcium spike following the Nod factor perception in the nodulation process. Calcium spiking, oscillations of the intracellular concentrations of Ca, is also an essential part of the signal transduction pathway required in nodule development [172], and therefore this gene is likely to play a significant role in nodule formation and subsequently, in N fixation efficiency.

## 5. Concluding Remarks

The natural diversity of rhizobia nodulating common bean has been widely studied, but, because of the promiscuity of this crop, novel symbionts of this legume should be expected and need to be screened. Information about rhizobia diversity in common bean serves to define host preferences and predominance of strains, to study the dynamics of exchange of genetic material, and provides a basis for the proposal of evolutionary trends. The diversity studies also reveal that each of the common bean-rhizobium associations coevolved independently after geographical separation, as did their genetic pools. Investigation about the structure of the indigenous rhizobial populations and their coevolution with the host plant could greatly contribute to better understanding and overcoming the frequent reports of nodulation failure. Molecular markers associated with nodulation genes are available in common bean, and QTL mapping studies showed that genes with varying effects seem to control N fixation. To date, few major QTLs and candidate genes have been reported in this legume. However, nodulating genes in model legumes have been cloned and several of their orthologs determined in common bean. Clearly, the evaluation of natural rhizobia diversity associated with common bean, making use of its well-characterized common bean biodiversity and feature-rich genomic tools, is becoming a powerful strategy of investigation, as are breeding cultivars for high symbiotic efficiency.

## References

[1] Grange L, Hungria M, Graham PH (2007). New insights in the origins and evolution of rhizobia that nodulate common bean (*Phaseolus vulgaris*) in Brazil. Soil Biol Biochem.

[2] CGIAR (Consultative Group on International Agricultural Research), Common Bean, 2012. http://www.cgiar.org/our-research/crops-factsheets/beans/.

[3] Aguilar OM, Lopez M, Riccillo PM (2001). The diversity of rhizobia nodulating beans in Northwest Argentina as a source of more efficient inoculant strains. J Biotechnol.

[4] Rodiño AP, Santalla M, De Ron AM, Lichtfouse E (2010). Co-evolution and migration of bean and rhizobia in Europe. Sociology, organic farming, climate change and soil science.

[5] Gepts P (1990). Biochemical evidence bearing on the domestication of *Phaseolus* beans. Econ Bot.

[6] Papa R, Nanni L, Sicard D (2006). The evolution of genetic diversity in *Phaseolus vulgaris* L. Darwin's harvest: New approaches to the origins, evolution and conservation of crops.

[7] Gepts P, Debouck D, van Schoonhoven A, Voysest O (1991). Origin, domestication and evolution of the common bean (*Phaseolus vulgaris* L.). Common Beans: Research for Crop Improvement.

[8] Santalla M, Rodiño AP, De Ron AM (2002). Allozyme evidence supporting Southwestern Europe as a secondary center of genetic diversity for the common bean. Theor Appl Genet.

[9] Gioia T, Logozzo G, Attene G (2013). Evidence for introduction bottleneck and extensive inter-gene pool (Mesoamerica × Andes) hybridization in the European common bean (*Phaseolus vulgaris* L.) Germplasm. PLoS One.

[10] Zeven AC (1997). The introduction of common bean (*Phaseolus vulgaris* L.) into Western Europe and the phenotypic variation of dry beans collected in the Netherlands in 1946. Euphytica.

[11] Oldroyd GED (2013). Speak, friend, and enter: signalling systems that promote beneficial symbiotic associations in plants. Nat Rev Microbiol.

[12] Olivares J, Bedmar EJ, Sanjuan J (2013). Biological nitrogen fixation in the context of global change. Mol Plant Microbe In.

[13] Galloway JN, Dentener FJ, Capone DG (2004). Nitrogen cycles: Past, present and future. Biogeochemistry.

[14] Bliss FA (1993). Breeding common bean for improved biological nitrogen fixation. Plant Soil.

[15] Fageria NK, Melo LC, Ferreira EPB (2014). Dry matter, grain yield, and yield components of dry bean as influenced by nitrogen fertilization and rhizobia. Commun Soil Sci Plant Anal.

[16] Hardarson G, Graham PH, Sadowsky MJ, Vance CP (1994). International FAO/IAEA programs on biological nitrogen fixation. Symbiotic Nitrogen Fixation.

[17] Graham PH (1981). Some problems of nodulation and symbiotic nitrogen fixation in *Phaseolus vulgaris*: a review. Field Crops Res.

[18] Piha MI, Munns DN (1987). Nitrogen-Fixation potential of beans (*Phaseolus vulgaris* L) compared with other grain legumes under controlled conditions. Plant Soil.

[19] Wolyn DJ, St Clair DA, DuBois J (1991). Distribution of nitrogen in common bean (*Phaseolus vulgaris* L.) genotypes selected for differences in nitrogen fixation ability. Plant Soil.

[20] Martínez-Romero E (2003). Diversity of *Rhizobium-Phaseolus vulgaris* symbiosis: overview and perspectives. Plant Soil.

[21] Miranda BD, Bliss FA (1991). Selection for increased seed nitrogen accumulation in common bean—implications for improving dinitrogen fixation and seed yield. Plant Breeding.

[22] Bonilla I, Bolaños L (2010). Mineral nutrition for Legume-Rhizobia Symbiosis: B, Ca, N, P, S, K, Fe, Mo, Co, and Ni: A Review. Organic Farming, Pest Control and Remediation of Soil Pollutants.

[23] Divito GA, Sadras VO (2014). How do phosphorous, potassium and sulphur affect plant growth and biological nitrogen fixation in crop and pasture legumes?. Field Crop Res.

[24] Muñoz-Azcárate O (2014). Sulphur metabolism in the pea-*Rhizobium* symbiosis, PhD Thesis Dissertation.

[25] Arrese-Igor C, Gonzalez EM, Marino D, Anjum NA, Lopez-Lauri F (2011). Physiological responses of legume nodules to drought. Plant Nutrition and Abiotic Stress Tolerance III, Plant Stress.

[26] Aranjuelo I, Arrese-Igor C, Molero G (2014). Nodule performance within a changing environmental context. J Plant Physiol.

[27] Bliss FA, Pereira PAA, Araujo RS (1989). Registration of 5 high nitrogen-fixing common bean germplasm lines. Crop Sci.

[28] De Souza EM, Bassani VL, Sperotto RA (2016). Inoculation of new rhizobial isolates improves nutrient uptake and growth of bean (*Phaseolus vulgaris*) and arugula (*Eruca sativa*). J Sci Food Agric.

[29] Sampaio FB, Knupp AM, Fernandes EP (2016). Morphophysiological characterization of rhizobia isolated from wild genotypes of the common bean. Bioscience J.

[30] Young JPW, Haukka K (1996). Diversity and phylogeny of rhizobia. New Phytol.

[31] Van Berkum P, Terefework Z, Paulin L (2003). Discordant phylogenies within the *rrn* loci of *Rhizobia*. J Bacteriol.

[32] Moussavi SA, Willems A, Nesme X (2015). Revised phylogeny of *Rhizobiaceae*: Proposal of the delineation of *Pararhizobium* gen. nov., and 13 new species combinations. Syst Appl Microbiol.

[33] Lindström K, Aserse AA, Mousavi SA, de Bruijn FJ (2013). Taxonomy and evolution of nitrogen-fixing organisms. Biological nitrogen fixation.

[34] Young JPW (2003). The genus name *Ensifer* Casida 1982 takes priority over *Sinorhizobium* Chen et al. 1988, and *Sinorhizobium morelense* Wang et al. 2002 is a later synonym of *Ensifer adhaerens* Casida 1982. Is the combination ‘*Sinorhizobium adhaerens*’ (Casida 1982) Willems et al. 2003 legitimate? Request for an Opinion. Int J Syst Evol Microbiol.

[35] CSP-Subcommittee on the taxonomy of Rhizobium and Agrobacterium—diversity, phylogeny and systematics. http://edzna.ccg.unam.mx/rhizobial-taxonomy/.

[36] Weir BS (2012). The current taxonomy of rhizobia. NZ Rhizobia website. http://www.rhizobia.co.nz/taxonomy/rhizobia.

[37] Valverde A, Igual JM, Peix A (2006). *R. lusitanum* sp. Nov a bacterium that nodulates *Phaseolus vulgaris*. Int J Syst Evol Microbiol.

[38] Martínez-Romero E, Segovia L, Mercante FM (1991). *Rhizobium tropici*, a novel species nodulating *Phaseolus vulgaris* L. beans and *Leucaena* sp tres. Int J Syst Bacteriol.

[39] Segovia L, Young JPW, Martínez-Romero E (1993). Reclassification of American *Rhizobium leguminosarum* biovar *phaseoli* type I strains as *Rhizobium etli* sp. Nov. Int J Syst Bacteriol.

[40] Amarguer N, Macheret V, Laguerre G (1997). *Rhizobium gallicum* sp. Nov., from *Phaseolus vulgaris* nodules. Int J Syst Bacteriol.

[41] Ribeiro RA, Martins TB, Ormeño-Orrillo E (2015). *Rhizobium ecuadorense* sp. nov., an indigenous N_2_-fixing symbiont of the Ecuadorian common bean (*Phaseolus vulgaris* L.) genetic pool. Int J Syst Evol Microbiol.

[42] Ribeiro RA, Rogel MA, López-López A (2012). Reclassification of *Rhizobium tropici* type A strains as *Rhizobium leucaenae* sp. nov. Int J Syst Evol Microbiol.

[43] Jordan DC, Krieg NR, Holt JG (1984). Family III Rhizobiaceae. Bergey's Manual of Systematic Bacteriology.

[44] Lopez-Lopez A, Rogel-Hernandez MA, Barois I (2012). *Rhizobium grahamii* sp. nov., from nodules of *Dalea leporina*, *Leucaena leucocephala* and *Clitoria ternatea*, and *Rhizobium mesoamericanum* sp. nov., from nodules of *Phaseolus vulgaris*, siratro, cowpea and *Mimosa pudica*. Int J Syst Evol Microbiol.

[45] Peng GX, Yuan QH, Li HX (2008). *Rhizobium oryzae* sp. nov., isolated from the wild rice *Oryza alta*. Int J Syst Evol Microbiol.

[46] Dall'Agnol RF, Ribeiro RA, Ormeno-Orrillo E (2013). *Rhizobium freirei* sp. nov., a symbiont of *Phaseolus vulgaris* that is very effective at fixing nitrogen. Int J Syst Evol Microbiol.

[47] Mnasri B, Liu TY, Saidi S (2014). *Rhizobium azibense* sp. nov., a nitrogen fixing bacterium isolated from root nodules of *Phaseolus vulgaris*. Int J Syst Evol Microbiol.

[48] Román-Ponce B, Zhang JY, Vásquez-Murrieta MS (2016). *Rhizobium acidisoli* sp. nov., isolated from root nodules of *Phaseolus vulgaris* in acid soils. Int J Syst Evol Microbiol.

[49] Yan J, Yan H, Liu LX (2017). *Rhizobium hidalgonense* sp. nov., a nodule endophitic bacterium of *Phaseolus vulgaris* in acid soil. Arch Microbiol.

[50] Mnasri B, Aouani ME, Mhamdi R (2007). Nodulation and growth of common bean (*Phaseolus vulgaris*) under water deficiency. Soil Biol Biochem.

[51] Zurdo-Piñeiro JL, García-Fraile P, Rivas (2009). Rhizobia from Lanzarote, the Canary Islands, that nodulate *Phaseolus vulgaris* have characteristics in common with *Sinorhizobium meliloti* isolates from mainland Spain. Appl Environ Microbiol.

[52] Mhamdi R, Laguerre G, Aouani ME (2002). Different species and symbiotic genotypes of field rhizobia can nodulate *Phaseolus vulgaris* in Tunisian soils. FEMS Microbiol Ecol.

[53] Toledo I, Lloret L, Martínez-Romero E (2003). *Sinorhizobium americanus* sp. nov., a new *Sinorhizobium* species nodulating native *Acacia* spp. in Mexico. Syst Appl Microbiol.

[54] Talbi C, Delgado MJ, Girard L (2010). *Burkholderia phymatum* strains capable of nodulating *Phaseolus vulgaris* are present in Moroccan soils. Appl Environ Microbiol.

[55] Aserse AA, Rasanen LA, Assefa F (2012). Phylogeny and genetic diversity of native rhizobia nodulating common bean (*Phaseolus vulgaris* L.) in Ethiopia. Syst Appl Microbiol.

[56] Martínez-Romero E, Caballero-Mellado J (1996). *Rhizobium* polygenies and bacterial genetic diversity. Crit Rev Plant Sci.

[57] Herrera-Cervera JA, Caballero-Mellado J, Laguerre G (1999). At least five rhizobial species nodulate *Phaseolus vulgaris* in a Spanish soil. FEMS Microbiol Ecol.

[58] Sessitsch A, Hardarson G, Akkermans ADL (1997). Characterization of *Rhizobium etli* and other *Rhizobium* spp. that nodulate *Phaseolus vulgaris* L. in an Austrian soil. Mol Ecol.

[59] Tjaholeksono A (1993). Caractérisation et diversité des souches de *Rhizobium* nodulant le haricot (*Phaseolus vulgaris* L.) cultivé en 3 sites tropicaux. Thesis dissertation.

[60] Diouf A, de Lajudie P, Neyra M (2000). Polyphasic characterization of rhizobia that nodulate *Phaseolus vulgaris* in West Africa (Senegal and Gambia). Int J Syst Evol Microbiol.

[61] Beyene D, Kassa S, Ampy F (2004). Ethiopian soils harbor natural populations of rhizobia that form symbioses with common bean (*Phaseolus vulgaris* L.). Arch Microbiol.

[62] Wang H, Man CX, Wang ET (2009). Diversity of rhizobia and interactions among the host legumes and rhizobial genotypes in an agricultural forestry ecosystem. Plant Soil.

[63] Lopez-Guerrero MG, Ormeño-Orrillo E, Velázquez E (2012). *Rhizobium etli* taxonomy revised with novel genomic data and analyses. Syst Appl Microbiol.

[64] Ribeiro RA, Ormeño-Orrillo E, Dall'Agnol RF (2013). Novel *Rhizobium* lineages isolated from root nodules of the common bean (*Phaseolus vulgaris* L.) in Andean and Mesoamerican areas. Res Microbiol.

[65] Mostasso FL, Dias BG, Vargas MAT (2002). Selection of bean (*Phaseolus vulgaris* L) rhizobial strains for the Brazilian Cerrados. Field Crops Res.

[66] Hungria M, Campo RJ, Mendes IC (2003). Benefits of inoculation of common bean (*Phaseolus vulgaris*) crop with efficienty and competitive *Rhizobium tropici* strains. Biol Fert Soils.

[67] Martínez-Romero E, Hernández-Lucas I, Peña-Cabriales JJ (1998). Symbiotic performance of some modified *Rhizobium etli* strains in assays with *Phaseolus vulgaris* beans that have a high capacity to fix N_2_. Plant Soil.

[68] Geniaux E, Flores M, Palacios R (1995). Presence of megaplasmids in *Rhizobium tropici* and further evidence of differences between the two *R. tropici* subtypes. Int J Syst Bacteriol.

[69] Hungria M, Andrade DD, Chueire LM (2000). Isolation and characterization of new efficient and competitive bean (*Phaseolus vulgaris* L.) rhizobia from Brazil. Soil Biol Biochem.

[70] Ormeño-Orrillo E, Menna P, Almeida LG (2012). Genomic basis of broad host range and environmental adaptability of *Rhizobium tropici* CIAT899 and *Rhizobium* sp. PRF 81 which are used in inoculants for common bean (*Phaseolus vulgaris* L.). BMC Genomics.

[71] Ormeño-Orrillo E, Gomes DF, Del Cerro P (2016). Genome of *Rhizobium leucaenae* strains CFN 299(T) and CPAO 29.8: searching for genes related to a successful symbiotic performance under stressful conditions. BMC Genomics.

[72] Silva C, Vinuesa P, Eguiarte LE (2003). *Rhizobium etli* and *Rhizobium gallicum* nodulate common bean (*Phaseolus vulgaris*) in a traditionally managed milpa plot in Mexico: Population genetics and biogeographic implications. Appl Environ Microbiol.

[73] Verástegui-Valdés MM, Zhang YJ, Rivera-Orduña FN (2014). Microsymbionts of *Phaseolus* vulgaris in acid and alkaline soils of Mexico. Syst Appl Microbiol.

[74] Bernal G, Graham PH (2001). Diversity in the rhizobia associated with *Phaseolus vulgaris* L. in Ecuador, and comparisons with Mexican bean rhizobia. Can J Microbiol.

[75] Miranda-Sánchez F, Rivera J, Vinuesa P (2016). Diversity patterns of *Rhizobiaceae* communities inhabiting soils, root surfaces and nodules reveal a strong selection of rhizobial partners by legumes. Environ Microbiol.

[76] Bitocchi E, Bellucci E, Giardini A (2013). Molecular analysis of the parallel domestication of the common bean (*Phaseolus vulgaris* L.) in Mesoamerica and the Andes. New Phytol.

[77] Valverde G, Otabbong E (1997). Evaluation of N_2_-fixation measured by the ^15^N-dilution and N-difference methods in Nicaraguan and Ecuadorian *Phaseolus vulgaris* L. plants inoculated with *Rhizobium leguminosarum* biovar. Acta Agr Scand B-S P.

[78] Kipe-Nolt JA, Montealegre CM, Tohme J (1994). Restriction of nodulation by the broad host range *Rhizobium tropici* strain CIAT899 in wild accessions of *Phaseolus vulgaris* L. New Phytol.

[79] Eardly BD, Wang F, Whittham TS (1995). Species limits in *Rhizobium* populations that nodulate the common bean (*Phaseolus vulgaris*). Appl Environ Microbiol.

[80] Aguilar OM, Lopez M, Riccillo PM (1998). Prevalence of the *Rhizobium etli*-like allele in genes coding for 16S rRNA among the indigenous populations associated with wild beans from the Southern Andes in Argentina. Appl Environ Microbiol.

[81] Aguilar OM, Riva O, Peltzer E (2004). Analysis of *R. etli* and of its symbiosis with wild *Phaseolus vulgaris* supports coevolution in centers of host diversification. Proc Natl Acad Sci USA.

[82] Grange L, Hungria M (2004). Genetic diversity of indigenous common bean (*Phaseolus vulgaris* L.) rhizobia in two Brazilian ecosystems. Soil Biol Biochem.

[83] Cardoso AA, Andraus MP, Oliveira TC (2017). Characterization of rhizobia isolates obtained from nodules of wild genotypes of common bean. Braz J Microbiol.

[84] Dall'Agnol RF, Ribeiro RA, Delamuta JR (2014). *Rhizobium paranaense* sp. nov., an effective N_2_-fixing symbiont of common bean (*Phaseolus vulgaris* L.) with broad geographical distribution in Brazil. Int J Syst Evol Microbiol.

[85] Díaz-Alcántara CA, Ramírez-Bahena MH, Mulas D (2014). Analysis of rhizobial strains nodulating *Phaseolus vulgaris* from Hispaniola Island, a geographic bridge between Meso and South America and the first historical link with Europe. Syst Appl Microbiol.

[86] Sánchez AC, Gutiérrez RT, Santana RC (2014). Effects of co-inoculation of native *Rhizobium* and *Pseudomonas strains* on growth parameters and yield of two contrasting *Phaseolus vulgaris* L. genotypes under Cuban soil conditions. Eur J Soil Biol.

[87] Urzúa H (2005). Beneficios de la fijación simbiótica de nitrógeno en Chile. Cien Inv Agr.

[88] Baginsky C, Brito B, Scherson R (2015). Genetic diversity of *Rhizobium* from nodulating beans grown in a variety of Mediterranean climate soils of Chile. Arch Microbiol.

[89] Bernal GR, Tlusty B, Estévez de Jensen C (2004). Characteristics of rhizobia nodulating beans in the central region of Minnesota. Can J Microbiol.

[90] Perez-Ramirez NO, Rogel MA, Wang E (1998). Seeds of *Phaseolus vulgaris* bean carry *Rhizobium etli*. FEMS Microbiol Ecol.

[91] Martínez E, Pardo MA, Palacios R (1985). Reiteration of nitrogen-fixation gene-sequences and specificity of *Rhizobium* in nodulation and nitrogen-fixation in *Phaseolus vulgaris*. J Gen Microbiol.

[92] Michiels J, Dombrecht B, Vermeiren N (1998). *Phaseolus vulgaris* is a non-selective host for nodulation. FEMS Microbiol Ecol.

[93] Valverde A, Velázquez E, Cervantes E (2011). Evidence of an American origin for Symbiosis-related genes in *Rhizobum lusitanum*. Appl Environ Microbiol.

[94] Velázquez A, Martínez-Romero E, Rodriguez-Navarro DN (2001). Characterization of rhizobial isolates of *Phaseolus vulgaris* by staircase electrophoresis of low-molecular-weight RNA. Appl Environ Microbiol.

[95] García-Fraile P, Mulas D, Peix A (2010). *Phaseolus vulgaris* is nodulated in Northern Spain by *Rhizobium leguminosarum* strains harboring two *nodC* alleles present in American *Rhizobium etli* strains: biogeographical and evolutionary implications. Can J Microbiol.

[96] Mulas D, García-Fraile P, Carro L (2011). Distribution and efficiency of *Rhizobium leguminosarum* strains nodulating *Phaseolus vulgaris* in Northern Spanish soils: Selection of native strains that replace conventional N fertilization. Soil Biol Biochem.

[97] Pohajda I, Babic KH, Rajnovic I (2016). Genetic diversity and symbiotic efficiency of indigenous common bean rhizobia in Croatia. Food Technol Biotech.

[98] Boumouch I, Brhada F, Filali-Maltouf A (2001). Selection of osmotolerant and effective strains of *Rhizobium* for inoculation of common bean (*Phaseolus vulgaris* L.) in Moroccan saline soils. Agronomie.

[99] Priefer UB, Aurag J, Boesten B (2001). Characterisation of *Phaseolus* symbionts isolated from Mediterranean soils and analysis of genetic factors related to pH tolerance. J Biotechnol.

[100] Faghire M, Mandri B, Oufdou K (2012). Identification at the species and symbiovar levels of strains nodulating *Phaseous vulgaris* in saline soils of the Marrakech region (Morocco) and analysis of the *otsA* gene putatively involved in osmotolerance. Syst Appl Microbiol.

[101] Mnasri B, Mrabet M, Laguerre G (2007). Salt-tolerant rhizobia isolated from a Tunisian oasis that are highly effective for symbiotic N_2_-fixation with *Phaseolus vulgaris* constitute a novel biovar (bv. *mediterranense*) of *Sinorhizobium meliloti*. Arch Microbiol.

[102] Mnasri B, Saïdi S, Chihaouri SA (2012). *Sinorhizobium americanum* symbiovar mediterranense is a predominant symbiont that nodulates and fixes nitrogen with common bean (*Phaseolus vulgaris* L.) in a Northern Tunisian field. Syst Appl Microbiol.

[103] Shamseldin AAY, Vinuesa P, Thierfelder H (2005). *Rhizobium etli* and *Rhizobium gallicum* nodulate *Phaseolus vulgaris* in Egyptian soils and display cultivar-dependent symbiotic efficiency. Symbiosis.

[104] Shamseldin A, Werner D (2005). High salt and high pH tolerance of new isolated *Rhizobium etli* strains from Egyptian soils. Curr Microbiol.

[105] Anyango B, Wilson KJ, Beynon JL (1995). Diversity of rhizobia nodulating *Phaseolus vulgaris* L. in two Kenyan soils with contrasting pHs. Appl Environ Microbiol.

[106] Kawaka F, Dida MM, Opala PA (2014). Symbiotic efficiency of native rhizobia nodulating common bean (*Phaseolus vulgaris* L.) in soils of Western Kenya. Int Sch Res Notices.

[107] Waswa NM (2014). Identifying elite rhizobia for commercial soybean (*Glycine max*) inoculants [MS Thesis].

[108] Zinga M, Jaiswat SK, Dakora FD (2017). Presence of diverse rhizobial communities responsible for nodulation of common bean (*Phaseolus vulgaris*) in South African and Mozambican soils. FEMS Microbiol Ecol.

[109] Gurkanli CT, Ozkoc I, Gunduz I (2013). Genetic diversity in rhizobia nodulating common bean (*Phaseolus vulgaris* L.) in the Central Black Sea Region of Turkey. Ann Microbiol.

[110] Tamimi SM, Young JPW (2004). *Rhizobium etli* is the dominant common bean nodulating rhizobia in cultivated soils from different locations in Jordan. Appl Soil Ecol.

[111] Assadi Rahmani H, Rasanen LA, Afshari A (2011). Genetic diversity and symbiotic effectiveness of rhizobia isolated from root nodules of *Phaseolus vulgaris* grown in soils of Iran. Appl Soil Ecol.

[112] Abbaszadeh-dahaji P, Savaghebi RG, Asadi-Rahmani H (2012). Symbiotic effectiveness and plant growth promoting traits in some Rhizobium strains isolated from *Phaseolus vulgaris* L. Plant Growth Regul.

[113] Zhang X, Blair MW, Wang S (2008). Genetic diversity of Chinese common bean (*Phaseolus vulgaris* L.) landraces assessed with simple sequence repeat markers. Theor Appl Genet.

[114] Van Scoonhoven A, Voyseste O (1991). Common beans: research for crop improvement.

[115] Han SZ, Wang ET, Chen WX (2005). Diverse bacteria isolated from root nodules of *Phaseolus vulgaris* and species withinthe genera *Campylotropis* and *Cassia* grown in China. Syst Appl Microbiol.

[116] Wang L, Cao Y, Wang ET (2016). Biodiversity and biogeography of rhizobia associated with common bean (*Phaseolus vulgaris* L.) in Shaanxi Province. Syst Appl Microbiol.

[117] Cao Y, Wang ET, Zhao L (2014). Diversity and distribution of rhizobia nodulated with *Phaseolus vulgaris* in two ecoregions of China. Soil Biol Biochem.

[118] Adhikari D, Itoh K (2013). Genetic diversity of common bean (*Phaseolus vulgaris* L.) nodulating rhizobia in Nepal. Plant Soil.

[119] Herridge DF, Redden RJ (1999). Evaluation of genotypes of navy and culinary bean (*Phaseolus vulgaris* L.) selected for superior growth and nitrogen fixation. Aust J Exp Agric.

[120] Kellman AW, Hill GD, McKenzie BA (2006). Is it worth inoculating common bean (*Phaseolus vulgaris* L.)?. Agronomy, NZ.

[121] Lafay B, Burdon JJ (2007). Molecular diversity of legume root-nodule bacteria in Kakadu National Park, Northern Territory, Australia. PLoS One.

[122] Eardly B, Elia P, Brockwell J (2017). Biogeography of a novel *Ensifer meliloti* clade associated with the Australian legume *Trigonella suavissima*. Appl Environ Microbiol.

[123] Young JPW, Johnston AWB (1989). The evolution of specificity in the legume-rhizobium symbiosis. Trends Ecol Evol.

[124] Wang D, Yang S, Tang F (2012). Symbiosis specificity in the legume-rhizobial mutualism. Cell Microbiol.

[125] Dalla Via V, Zanetti ME, Blanco F (2016). How legumes recognize rhizobia. Plant Signal Behav.

[126] Cooper JE (2007). Early interactions between legumes and rhizobia: disclosing complexity in a molecular dialogue. J Appl Microbiol.

[127] Janczarek M (2011). Environmental signals and regulatory pathways that influence exopolysaccharide production in hizobia. Int J Mol Sci.

[128] Sutherland IW (1972). Bacterial exopolysaccharides. Adv Microb Physiol.

[129] Gibson KE, Kobayashi H, Graham C (2008). Molecular determinants of a symbiotic chronic infection. Annu Rev Genet.

[130] Long SR (1996). Rhizobium symbiosis: Nod factors in perspective. Plant Cell.

[131] Mergaert P, Van Montagu M, Holsters M (1997). Molecular mechanisms of Nod factor diversity. Mol Microbiol.

[132] Bolaños-Vásquez MC, Werner D (1997). Effect of *Rhizobium tropici*, *R. etli*, and *R. leguminosarum* bv. *phaseoli* on nod gene inducing flavonoids in root exudates of *Phaseolus vulgaris*. Mol Plant Microbe In.

[133] Martínez-Romero E (2009). Coevolution in Rhizobium-legume symbiosis?. DNA Cell Biol.

[134] Jorrín B, Imperial J, de Brujin FJ (2015). Pool-seq analysis of microsymbiont selection by the legume plant host. Biological Nitrogen Fixation.

[135] Jorrín B, Imperial J (2015). Population genomics analysis of legume host preference for specific rhizobial genotypes in the *Rhizobium leguminosarum* bv. *viciae* symbioses. Mol Plant Microbe In.

[136] Reid DE, Ferguson BJ, Hayashi S (2011). Molecular mechanisms controlling legume autoregulation of nodulation. Ann Bot.

[137] Formey D, Martín-Rodríguez JÁ, Leija A (2016). Regulation of small RNAs and corresponding targets in Nod factor-induced *Phaseolus vulgaris* root hair cells. Int J Mol Sci.

[138] Vargas C, Martínez LJ, Megías M (1990). Identification and cloning of nodulation genes and host specificity determinants of the broad host-range *Rhizobium leguminosarum* strain CIAT899. Mol Microbiol.

[139] Yang GP, Debellé F, Savagnac A (1999). Structure of the *Mesorhizobium hualuii* and *Rhizobium galegae* Nod factors: a cluster of phylogenetically related legumes are nodulated by rhizobia producing Nod factors with α, β-unsaturated N-acyl substitutions. Mol Microbiol.

[140] Soria-Díaz ME, Rodríguez-Carvajal MA, Tejero-Mateo P (2006). Structural determination of the Nod factors produced by *Rhizobium gallicum* bv. *gallicum* R602. FEMS Microbiol Lett.

[141] Del Cerro P, Rolla-Santos AA, Gomes DF (2015). Opening the “black box” of *nodD3*, *nodD4* and *nodD5* genes of *Rhizobium tropici* strain CIAT899. BMC Genomics.

[142] Del Cerro P, Rolla-Santos AA, Gomes DF (2015). Regulatory *nodD1* and *nodD2* genes of *Rhizobium tropici* strain CIAT899 and their roles in the early stages of molecular signaling and host-legume nodulation. BMC Genomics.

[143] Prudent M, Salon C, Smith DL (2016). Nod factor supply under water stress conditions modulates cytokinin biosynthesis and enchances nodule formation and N nutrition in soybean. Plant Signal Behav.

[144] Lafuente A, Pérez-Palacios P, Doudkali B (2015). Unraveling the effect of arsenic on the model *Medicago-Ensifer* interaction: a transcriptomic meta-analysis. New Phytol.

[145] Duzan HM, Zhou X, Souleimanov A (2004). Perception of *Bradyrhizobium japonicum* Nod factor in soybean [Glycine max (L.) Merr.] root hairs under abiotic stress conditions. J Exp Bot.

[146] de Souza EM, Granada CE, Sperotto RA (2016). Plant pathogens affecting the establishment of Plant-Symbiont Interaction. Front Plant Sci.

[147] Perez-Carrascal OM, Vanlsberghe D, Juarez S (2016). Population genomics of the symbiotic plasmids of sympatric nitrogen-fixing Rhizobium species associated with *Phaseolus vulgaris*. Environ Microbiol.

[148] Laguerre G, Nour SM, Macheret V (2001). Classification of rhizobia based on *nodC* and *nifH* gene analysis reveals a close phylogenetic relationship among *Phaseolus vulgaris* symbionts. Microbiology.

[149] Cervantes L, Bustos P, Girard L (2011). The conjugative plasmid of a bean-nodulating Sinorrhizobium fredii strain is assembled from sequences of two *Rhizobium* strains and the chromosome of a Sinorhizobium strain. BMC Microbiol.

[150] Resendis-Antonio O, Hernandez M, Salazae E (2011). System biology of bacterial nitrogen fixation: high throughput technology and its integrative description with constraint-based modeling. BMC Syst Biol.

[151] Gomes DF, Batista JS, Schiavon AL (2012). Proteomic profiling of *Rhizobium tropici* PRF 81: identification of conserved and specific responses to heat stress. BMC Microbiol.

[152] Limpens E, Franken C, Smit P (2003). LysM domain receptor kinases regulating rhizobial Nod factor-induced infection. Science.

[153] Sato S, Nakamura Y, Kaneko T (2008). Genome structure of the legume *Lotus japonicas*. DNA Res.

[154] Young ND, Debelle F, Oldroyd GE (2011). The Medicago genome provides insight into the evolution of rhizobial symbioses. Nature.

[155] Schmutz J, Cannon SB, Schlueter J (2010). Genome sequence of the palaeopolyploid soybean. Nature.

[156] Varshney RK, Song C, Saxena RK (2013). Draft genome sequence of chickpea (*Cicer arietinum*) provides a resource for trait improvement. Nat Biotechnol.

[157] Schmutz J, McLean P, Mamidi S (2014). A reference genome for common bean and genome-wide analysis of dual domestications. Nat Genet.

[158] Vlasova A, Capella-Gutierrez S, Rendon-Anaya M (2016). Genome and transcriptome analysis of the Mesoamerican common bean and the role of gene duplications in establishing tissue and temporal specialization of genes. Genome Biol.

[159] Lin JY, Stupar RM, Hans C (2010). Structural and functional divergence of a 1-Mb duplicated region in the soybean (*Glycine max*) genome and comparison to an orthologous region from *Phaseolus vulgaris*. Plant Cell.

[160] McClean PE, Marnidi S, McConnell M (2010). Synteny mapping between common bean and soybean reveals extensive blocks of shared loci. BMC Genomics.

[161] Schlueter JA, Dixon P, Granger C (2004). Mining EST databases to resolve evolutionary events in major crop species. Genome.

[162] Kalavacharia V, Liu Z, Meyers BC (2011). Identification and analysis of common bean (*Phaseolus vulgaris* L.) transcriptomes by massively parallel pyrosequencing. BMC Plant Biol.

[163] Endre G, Kereszt A, Kevei Z (2002). A receptor kinase gene regulating symbiotic nodule development. Nature.

[164] Zhu H, Choi HK, Cook DR (2005). Bridging model and crop legumes through comparative genomics. Plant Physiol.

[165] Kim DH, Parupalli S, Azam S (2013). Comparative sequence analysis of nitrogen fixation-related genes in six legumes. Front Plant Sci.

[166] Qiao Z, Pingault L, Nourbakhsh-Rey M (2016). Comprehensive comparative genomic and transcriptomic analyses of the legume genes controlling the nodulation process. Front Plant Sci.

[167] Ferguson BJ, Indrasumunar A, Hayashi S (2010). Molecular analysis of legume nodule development and autorregulation. J Integr Plant Biol.

[168] Shritliffe SJ, Vessey JK, Buttery (1996). Comparison of growth and N accumulation of common bean (*Phaseolus vulgaris* L.), cv. OAC Rico and its two nodulation mutants, R69 and R99. Can J Plant Sci.

[169] Abd-Alla MH (2011). Nodulation and nitrogen fixation in interspecies grafts of soybean and common bean is controlled by isoflavonoid signal molecules translocated from shoot. Plant Soil Environ.

[170] Hastwell AH, Gresshoff PM, Ferguson BJ (2015). Genome-wide annotation and characterization of CLAVATA/ESR (CLE) peptide hormones of soybean (*Glycine max*) and common bean (*Phaseolus vulgaris*), and their orthologues of *Arabidopsis thaliana*. J Exp Bot.

[171] Ferguson BJ, Mathesius U (2014). Phytohormone regulation of legume-rhizobia interactions. J Chem Ecol.

[172] Oldroyd GED, Downie JA (2008). Coordinating nodule morphogenesis with rhizobial infection in legumes. Annu Rev Plant Biol.

[173] Reid DE, Ferguson BJ, Gresshoff PM (2011). Inoculation- and nitrate-induced CLE peptides of soybean control NARK-dependent nodule formation. Mol Plant Microbe In.

[174] O'Rourke JA, Iniguez LP, Fu F (2014). An RNA-Seq based gene expression atlas of the common bean. BMC Genomics.

[175] Carroll BJ, McNeil DL, Gresshoff PM (1985). Isolation and properties of soybean [*Glycine max* (L.) Merr.] mutants that nodulate in the presence of high nitrate concentrations. Proc Natl Acad Sci USA.

[176] Carroll BJ, McNeil DL, Gresshoff PM (1985). A supernodulation and nitrate tolerant symbiotic (nts) soybean mutant. Plant Physiol.

[177] Gresshoff PM (1993). Molecular genetic analysis of nodulation genes in soybean. Plant Breed Rev.

[178] Szczyglowski K, Shaw RS, Wopereis J (1998). Nodule organogenesis and symbiotic mutants of the model legume *Lotus japonicus*. Mol Plant Microbe In.

[179] Zanetti ME, Blanco FA, Beker MP (2010). A C subunit of the plant nuclear factor NF-Y required for rhizobial infection and nodule development affects partner selection in the common bean-*Rhizobium etli* symbiosis. Plant Cell.

[180] Ramírez M, Graham MA, Blanco-López L (2005). Sequencing and analysis of common bean ESTs: Building a foundation for functional genomics. Plant Physiol.

[181] Peltzer Meschini EP, Blanco FA, Zanetti ME (2008). Host genes involved in nodulation preference in common bean (*Phaseolus vulgaris*)-*Rhizobium etli* symbiosis revealed by suppressive subtractive hybridization. Mol Plant Microbe In.

[182] Galeano CH, Cortés AJ, Fernandez AC (2012). Gene-based single nucleotide polymorphism markers for genetic and association mapping in common bean. BMC Genet.

[183] Quiceno-Rico JM, Camas-Reyes JL, Alvarez-Venegas R (2012). Molecular cloning and characterization of two trithorax-group genes from *Phaseolus vulgaris* roots and symbiotic nodules. Plant Omics.

[184] Montiel J, Arthikala MK, Quinto C (2013). *Phaseolus vulgaris*
*RbohB* functions in lateral root development. Plant Signal Behav.

[185] Islas-Flores T, Guillén G, Alvarado-Affantranger X (2011). PvRACK1 loss-of-function impairs expansion and morphogenesis in *Phaseolus vulgaris* L. root nodules. Mol Plant Microbe In.

[186] Barraza A, Estrada-Navarrete G, Rodriguez-Algeria ME (2013). Down-regulation of PvTRE1 enhances nodule biomass and bacteriod number in the common bean. New Phytol.

[187] Dalla Via V, Narduzzi C, Aguilar OM (2015). Changes in the common bean transcriptome in response to secreted and surface signal molecules of *Rhizobium etli*. Plant Physiol.

[188] Yan Z, Hossain MS, Wang J (2013). miR172 regulates soybean nodulation. Mol Plant Microbe In.

[189] Wang Y, Wang L, Zou Y (2014). Soybean miR172c targets the repressive AP2 transcription factor NNC1 to activate ENOD40 expression and regulate nodule initiation. Plant Cell.

[190] Nova-Franco B, Íñiguez LP, Valdés-López O (2015). The miR172c-AP2-1 node as a key regulator of the common bean-rhizobia nitrogen fixation symbiosis. Plant Physiol.

[191] Buttery BR, Park SJ, Berkum PV (1997). Effects of common bean (*Phaseolus vulgaris* L) cultivar and *Rhizobum* strain on plant growth, seed yield and nitrogen content. Can J Plant Sci.

[192] Elizondo Barron J, Pasino RJ, Davis DW (1999). Response to selection for seed yield and nitrogen (N_2_) fixation in common bean (*Phaseolus vulgaris* L.). Field Crops Res.

[193] Graham PH, Rosas J (1977). Growth and development of indeterminate bush and climbing cultiuvars of *Phaseolus vulgaris* L. inoculated with *Rhizobium*. J Agric Sci.

[194] Pereira PAA, Miranda BD, Attewell JR (1993). Selection for increased nodule number in common bean (*Phaseolus vulgaris* L.). Plant Soil.

[195] Rennie RJ, Kemp GA (1983). N_2_-fixation in field beans quantified by ^15^N isotope dilution. II. Effect of cultivars of beans. Agron J.

[196] Buttery BR, Park SJ, van Berkum P (1997). Effects of common bean (*Phaseolus vulgaris* L.) cultivar and rhizobium strain on plant growth, seed yield and nitrogen content. Can J Plant Sci.

[197] Rodiño AP, De La Fuente M, De Ron AM (2011). Variation for nodulation and plant yield of common bean genotypes and environmental effects on the genotype expression. Plant Soil.

[198] Rodiño AP, Santalla M, De Ron AM (2005). variability in symbiotic nitrogen fixation among white landraces of common bean from the Iberian peninsula. Symbiosis.

[199] Polania J, Poschenrieder C, Rao I (2016). Estimation of phenotypic variability in symbiotic nitrogen fixation ability in common bean under drought stress using ^15^N natural abundance in grain. Eur J Agron.

[200] Nodari RO, Tsai SM, Guzman P (1993). Towards an integrated linkage map of common bean III: Mapping genetic factors controlling host-bacteria interactions. Genetics.

[201] Tsai S, Nodari R, Moon D (1998). QTL mapping for nodule number and common bacterial blight in *Phaseolus vulgaris* L. Plant Soil.

[202] Souza A, Boscariol R, Moon D (2000). Effects of *Phaseolus vulgaris* QTL in controlling host-bacteria interactions under two levels of nitrogen fertilization. Gen Mol Biol.

[203] Ramaekers L, Galeano C, Garzón N (2013). Identifying quantittative trait loci for symbiotic nitrogen fixation capacity and related traits in common bean. Mol Breeding.

[204] Kamfwa K, Cichy KA, Kelly JD (2015). Genome-wide association analysis of symbiotic nitrogen fixation in common bean. Theor Appl Genet.

[205] Dwivedi SL, Sahrawat KL, Upadhyaya HD (2015). Advances in host plant and rhizobium genomics to enhance symbiotic nitrogen fixation in grain legumes. Adv Agron.

[206] Franco MC, Cassini ST, Oliveira VR (2001). Combining ability for nodulation in common bean (Phaseolus vulgaris L.) genotypes from Andean and middle American gene pools. Euphytica.

[207] Asfaw A, Blair MW, Struik P (2012). Multi-environment quantitative trait loci analysis for photosynthate acquisition, accumulation, and remobilization traits in common bean under drought stress. G3 Genes Genom Genet.

[208] Farid M (2015). Symbiotic nitrogen fixation in common bean. PhD Thesis Dissertation.

[209] Heilig JA, Beaver JS, Wright EM (2017). QTL analysis of symbiotic nitrogen fixation in a black bean population. Crop Sci.

[210] Sánchez-López, Jáuregui D, Nava N (2011). Down-regulation of *SymRK* correlates with a deficiency in vascular bundle development in *Phaseolus vulgaris* nodules. Plant Cell Environ.

[211] Mitra RM, Gleason CA, Edwards A (2004). A Ca2+/calmodulin-dependent protein kinase required for symbiotic nodule development: gene identification by transcript-based cloning. Proc Natl Acad Sci USA.

[212] Pedalino MJ, Kipe-Nolt L (1993). Common bean (*Phaseolus vulgaris* L.) mutants defective in root nodule formation. I. Physiological characterization. J Exp Bot.

[213] Pedalino MJ, Kipe-Nolt L, Frusciante L (1993). Common bean (*Phaseolus vulgaris* L.) mutants defective in root nodule formation. II. Genetic analysis. J Exp Bot.

[214] Park SJ, Buttery BR (1988). Nodulation mutants of white bean (*Phaseolus vulgaris* L.), induced by ethyl-methane sulphonate. Can J Plant Sci.

[215] Park SJ, Buttery BR (1997). Complementation of nodulation genes of various mutants in common bean (*Phaseolus vulgaris* L.). J Hered.

[216] Pedalino M, Giller KE, Kipe-Nolt J (1992). Genetics of physiological characterization of non-nodulating mutant of *Phaseolus vulgaris* L.-NOD125. J Exp Bot.

[217] Park SJ, Buttery BR (1989). Identiﬁcation and characterization of common bean (*Phaseolus vulgaris* L.) lines well nodulated in the presence of high nitrate. Plant Soil.

[218] Park SJ, Buttery BR (1994). Inheritance of non-nodulation and ineffective nodulation mutants in common bean. J Hered.

[219] Shritliffe SJ, Vessey JK (1996). A nodulation (Nod+/Fix-) mutant of *Phaseolus vulgaris* L. has nodule-like structures lacking peripheral vascular bundles (Pvb-) and is resistant to mycorrhizal infection (Myc-). Plant Sci.

